# Novel insights on demographic history of tribal and caste groups from West Maharashtra (India) using genome-wide data

**DOI:** 10.1038/s41598-020-66953-3

**Published:** 2020-06-22

**Authors:** Guilherme Debortoli, Cristina Abbatangelo, Francisco Ceballos, Cesar Fortes-Lima, Heather L. Norton, Shantanu Ozarkar, Esteban J. Parra, Manjari Jonnalagadda

**Affiliations:** 1 Department of Anthropology, University of Toronto at Mississauga, Mississauga, ON Canada; 2 Department of Biological Sciences, Middle East Technical University, Çankaya, Turkey; 3 Sub-department of Human Evolution, Department of Organismal Biology, Evolutionary Biology Centre, Uppsala University, Uppsala, Sweden; 4 Department of Anthropology, University of Cincinnati, Cincinnati, OH USA; 5 Department of Anthropology, Savitribai Phule Pune University, Pune, India; 6 Symbiosis School for Liberal Arts, Symbiosis International (Deemed University), Pune, India

**Keywords:** Anthropology, Population genetics, Evolution

## Abstract

The South Asian subcontinent is characterized by a complex history of human migrations and population interactions. In this study, we used genome-wide data to provide novel insights on the demographic history and population relationships of six Indo-European populations from the Indian State of West Maharashtra. The samples correspond to two castes (Deshastha Brahmins and Kunbi Marathas) and four tribal groups (Kokana, Warli, Bhil and Pawara). We show that tribal groups have had much smaller effective population sizes than castes, and that genetic drift has had a higher impact in tribal populations. We also show clear affinities between the Bhil and Pawara tribes, and to a lesser extent, between the Warli and Kokana tribes. Our comparisons with available modern and ancient DNA datasets from South Asia indicate that the Brahmin caste has higher Ancient Iranian and Steppe pastoralist contributions than the Kunbi Marathas caste. Additionally, in contrast to the two castes, tribal groups have very high Ancient Ancestral South Indian (AASI) contributions. Indo-European tribal groups tend to have higher Steppe contributions than Dravidian tribal groups, providing further support for the hypothesis that Steppe pastoralists were the source of Indo-European languages in South Asia, as well as Europe.

## Introduction

The South Asian subcontinent is characterized by a complex history of human migrations and interactions, as well as a variety of traditional practices, all of which have contributed to an extensive cultural and genetic diversity. One of the defining characteristics of the Indian human landscape is the presence of both tribal and non-tribal groups^1^. Tribal populations are considered to be the indigenous populations of India and constitute approximately 8.63% of the total population^2^. The non-tribal populations consist of religious communities outside the caste system, and hierarchically arranged, endogamous social groups known as castes^3^. This fundamental division of the Indian population into castes, tribes and religious groups has resulted in a large number of effective populations or endogamous groups^1,3^.

India’s genetic diversity can be best described by a model of mixture between two statistically reconstructed ancestral populations: The Ancestral Northern Indians (ANI), which are related genetically to West Eurasians, including Middle Easterners, Central Asians and Europeans, and Ancestral South Indians (ASI), which are distantly related to Indigenous Andaman Islanders^4^. ANI ancestry is proportionally higher among Indo-European speakers and is also more prevalent in upper castes than in lower or middle castes^4^. Furthermore, the ANI component has a discernable geographic pattern in South Asia decreasing from the northwest. This gradient relating the ANI with the ASI is known as the “Indian Cline”^4^.

Recent studies incorporating genomic data from both ancient DNA (aDNA) and present-day South Asians have modeled the formation of the Indian Cline as a combination of three source populations: (1) the Ancient Ancestral South Indians (AASI), which represent a hypothesized South Asian Hunter-Gatherer lineage arising from a population split contemporaneous with the split of East Asian, Onge and Australian Aboriginal Ancestors; (2) Ancient Iranians and (3) Middle and Late Bronze Age Steppe populations (Steppe-MLBA)^5–7^. These studies suggest that a distinctive genetic mixture of ancient Iranians and AASI could have formed the genetic basis of the Indus Valley Civilization (IVC), thus this mixture was denoted the “Indus Periphery”. It has been hypothesized that the Indus Periphery played a pivotal role in the transformation of the three source populations into the two ancestral populations, ANI and ASI, which shape the current Indian Cline^5,6^. During the decline of the IVC, a portion of the Indus Periphery population admixed with the AASI to form the ASI in the Southern regions of India, whereas subsequent interaction between Indus Periphery and incoming Steppe-MLBA populations in the Northern regions resulted in the formation of the ANI^5,6^. Notably, following this period of admixture (4200-1900 years ago), Indian populations appear to have exhibited a shift toward endogamy, thereafter reducing gene flow^8^. In this context, a range of historic migrations and long-standing socio-cultural divisions have structured India’s genetic variation into a unique pattern of different endogamous groups.

This study presents genome-wide data from six modern populations from the Indian State of West Maharashtra (WM). The samples correspond to two castes (Deshastha Brahmins and Kunbi Marathas,) and four tribal groups (Kokana, Warli, Bhil and Pawara), all of which belong to the Indo-European language family^9^. These samples were analyzed together with previously reported South Asian modern and ancient samples to infer their population-structure and demographic history.

## Methods

### Sampling procedures

The study participants included unrelated individuals representing four tribal populations (Kokana, Warli, Bhil and Pawara) and two caste populations (Deshastha Brahmins and Kunbi Marathas) collected from West Maharashtra, India. Sampling of tribal populations was done from Jawhar (19.91°N, 73.23°E) in the Palghar district and Dhadgaon (21.82°N, 74.22°E) in the Nandurbar district of West Maharashtra. Caste populations were sampled from the city of Pune and adjoining villages (18.53°N, 73.87°E) using purposive sampling (Neyman, 1934). Authors MJ and SO travelled to these communities and advertised the study in the community looking for volunteers. Healthy individuals from both genders with no apparent skin disorders were included in the study. Details of age, sex, birthplace and clan were recorded using a questionnaire, and a sample of whole blood (5-8 mL) was collected in EDTA vials from each study participant after obtaining informed written consent. DNA was extracted using the phenol-chloroform extraction method^10^, and quantified using the EPPENDORF Biophotometer Plus. All methods of data collection were conducted as per ethical guidelines and regulations after receiving approval from the Institutional Ethics Committee (IEC) at the Savitribai Phule Pune University (Ethics/2012/16).

### Genotyping

Genotyping was carried out for 480 DNA samples with the APPLIED BIOSYSTEM’S AXIOM Precision Medicine Research Array (PMRA) at the Imperial Life Sciences Pvt Ltd. Laboratory (Gurgaon, Haryana, India) using standard protocols. The PMRA array includes approximately 900,000 markers and was designed to capture common genome variation in diverse population groups. The program Axiom Analysis Suite was used to carry out basic quality-control (QC) steps. After these initial QC steps, 522,125 polymorphic markers and 478 samples were retained for further analyses.

We performed additional QC steps to remove samples and markers. Briefly, we filtered out: 1) samples with sex discrepancies 2) samples that were outliers for heterozygosity, 3) samples with missing call rates <0.95, 4) related individuals (pi-hat> 0.25), and 5) samples that were outliers in Principal Component Analysis (PCA) plots. We also removed: 1) markers with genotype call rate <0.95, 2) markers with Hardy-Weinberg (HW) p-values <10^-6^, 3) markers with minor allele count <4, 4) Insertion/Deletion (Indel) markers, 5) markers not present in the 1000 Genomes reference panel, or that did not match on chromosome, position and alleles, 6) A/T or G/C SNPs with MAF > 40% in the 1000 Genomes South Asian reference samples, and 7) SNPs with allele frequency differences> 20% between the study sample and the 1000 Genomes South Asian reference sample. After these QC steps, we retained 456 samples and 398,118 autosomal markers. The samples were phased using the program SHAPEIT2^11^.

### Identification of runs of homozygosity

ROH were called using PLINK v1.9^12^. The PLINK conditions applied were based on previous studies^13,14^:*–homozyg-snp 30;–homozyg-kb 300;–homozyg-density 30;–homozyg-window-snp 30;–homozyg-gap 1000;–homozyg-window-het 1;–homozyg-window-missing 5;–homozyg-window-threshold 0.05*. A SNP coverage of 504 K for all the populations analysed enables a single SNP to be found, on average, in a track of 5.7 Kb. To obtain a window with 30 SNPs, on average (assuming a homogenous SNP distribution along the genome), a track of 171 Kb is needed. A threshold of 300 Kb was set for the minimum length in order to capture small ROH that originated far in the past and also to ensure that these are true ROH that arose by genetic drift or consanguinity. An alternative source of homozygosity originates from linkage disequilibrium (LD), which typically produces tracts measuring up to 100 Kb, based on empirical studies^15,16^. By using a minimum-length cut-off of 300 Kb, most short ROH resulting from LD are removed.

### Uncovering the source of Inbreeding

Inbreeding can be produced by two different biological phenomena: deviation from panmixia, in what G. Malecot called systematic inbreeding^17^, or by genetic drift and low effective population size, also called panmictic inbreeding^18^. The two different sources of inbreeding, namely, systematic inbreeding or non-random mating (F_IS_), and genetic drift (denoted by F_ST_), are both components of the total inbreeding coefficient (F_IT_). F_IT_ is defined as the probability that an individual receives two alleles that are identical-by-descent. Sewall Wright developed an approach to consider these three different F coefficients in his F statistics (1 − F_IT_) = (1 − F_IS_)(1 − F_ST_)^19^. First defined as correlations, Nei showed how these coefficients can be expressed in terms of allele frequencies, and observed and expected genotype frequencies^20,21^. Systematic inbreeding has a direct effect on the HW proportions of a population and can be measured using the Wright’s fixation index or F_IS_^19^. In this context F_IS_ is the average SNP homozygosity within an individual relative to the expected homozygosity of alleles randomly drawn from the population. This component of total inbreeding coefficient is measured using the*–het* function in PLINK. Panmictic inbreeding due to genetic drift (F_ST_) can be considered a measure of the genetic differentiation of a subpopulation in comparison with an ideal population with a large N_e_. F_IT_ is the total inbreeding coefficient, traditionally obtained using deep genealogies, and can be calculated using the sum of Runs Of Homozygosity (ROH) higher than 1.5 Mb^22^.

### Identity by descent analyses

To investigate identity-by-descent (IBD) segments in the phased genotype data we used the Refined IBD program (12Jul18 version)^23^, defining 2 cM as the minimum length for reporting IBD segments. Default settings of the program were applied for all other remaining parameters. After detecting the IBD segments with Refined IBD, we removed breaks and short gaps in IBD segments using the merge-ibd-segments java program (12Jul18 version). Following authors’ recommendations, we removed gaps between IBD segments that had at most one discordant homozygote and that were less than 0.6 cM in length. Networks of individuals were generated based on their shared IBD lengths using Gephi software^24^. Each node represents an individual, and the length of an edge equals to 1/(total shared IBD). Therefore, in these networks, individuals sharing more IBD segments are located closer than individuals with limited IBD sharing. The IBDNe program^25^ was used to estimate effective population sizes in the last 50 generations, applying the default settings of the program.

### Merging samples with other datasets

To obtain additional insights about the demographic history of our WM samples, we merged the WM database with other available data from both modern and ancient samples. We first merged the genotype data for markers without missing data in the WM sample (~126 K markers) with data for South Asian samples of the 1000 Genomes Project^26^ and the Simons Genome Diversity Project (SGDP)^27^, hereinafter referred to as the WM-1KG-SGDP dataset. We then merged the WM-1KG-SGDP dataset with ancient DNA data generated by Narasimhan *et al*.^5^. After merging, the number of autosomal markers in the WM-1KG-SGDP-aDNA dataset was ~107 K markers.

### Population structure analyses

PCA analyses based on LD-pruned markers were performed using the smartpca algorithm implemented in EIG v7.2.1^28,29^.For the WM-1KG-SGDP PCA, the number of markers after LD-pruning with PLINK 1.9 (r2 = 0.1) was ~73 K. For the WM-1KG-SGDP-aDNA PCA we only included markers with genotyping rates higher than 80%, and samples with genotyping rates higher than 80% (except for one ancient Indus Periphery sample that had a genotyping rate higher than 60%). The final number of markers after LD-pruning with PLINK 1.9 (r2 = 0.2) was ~70 K (genotyping rate = 95.9%). Ancient DNA samples were projected onto the PCA plot generated with the modern samples, using the *lsqproject* = *YES* and *shrinkmode* = *YES* options of smartpca. Another PCA analysis was also carried out without projecting the aDNA samples. Unsupervised clustering of the samples included in the LD-pruned WM-1KG-SGDP-aDNA dataset was carried out with the program ADMIXTURE^30^.

### Allele sharing analyses and admixture models

The ADMIXTOOLS bundle implemented in the R package admixr^31^ was used to carry out allele sharing analyses with the f3 and f4 statistics^32^ based on the WM-1KG-SGDP-aDNA dataset. First, we calculated the outgroup f3 statistic, which measures the amount of shared genetic drift between two South Asian populations using an African population (Mbuti) as an outgroup with the topology f3(Mbuti; A, B). Second, we applied f4 statistics to test for admixture in modern South Asian populations. For putative parental groups for this test, we selected the following samples based on our PCA and ADMIXTURE estimates: Ganj-Dareh (Iranian Agriculturalists), Steppe-MLBA (Steppe population) and Andamanese (AASI population). For these tests, we also used Mbuti as an outgroup, using the topology f4 (Outgroup,X:A,B). Negative values indicate that there is more allele sharing between population X and population A than between the population X and B. Positive values indicate that there is more allele sharing between population X and B than between population X and A. The significance of the f3 and f4 statistics is based on a Z-score calculated by block jackknife. Finally, we used the program qpAdm^33^ to estimate the relative contributions of AASI-related, Indus-Diaspora-related and Steppe-related groups to the WM tribal and caste populations, as well as other relevant populations from South Asia. For this analysis we merged the WM-1KG-SGDP-aDNA dataset with genotypes of present-day and ancient DNA data, resulting in a sample file with ~52 K markers. For this test we used the same samples as Narasimhan *et al*.^5^, i. e., Onge, Steppe-MLBA and Indus Periphery as source groups (representing the AASI-related group, the Steppe pastoralist group and the ancient Iranian group), and Mbuti, Iron_Gates_HG, Karelia_HG, Ganj_Dareh_N, Anatolia_N, West_Siberia_N, Han, and Karitiana as outgroups.

## Results

### ROH analyses

**Figure 1:**
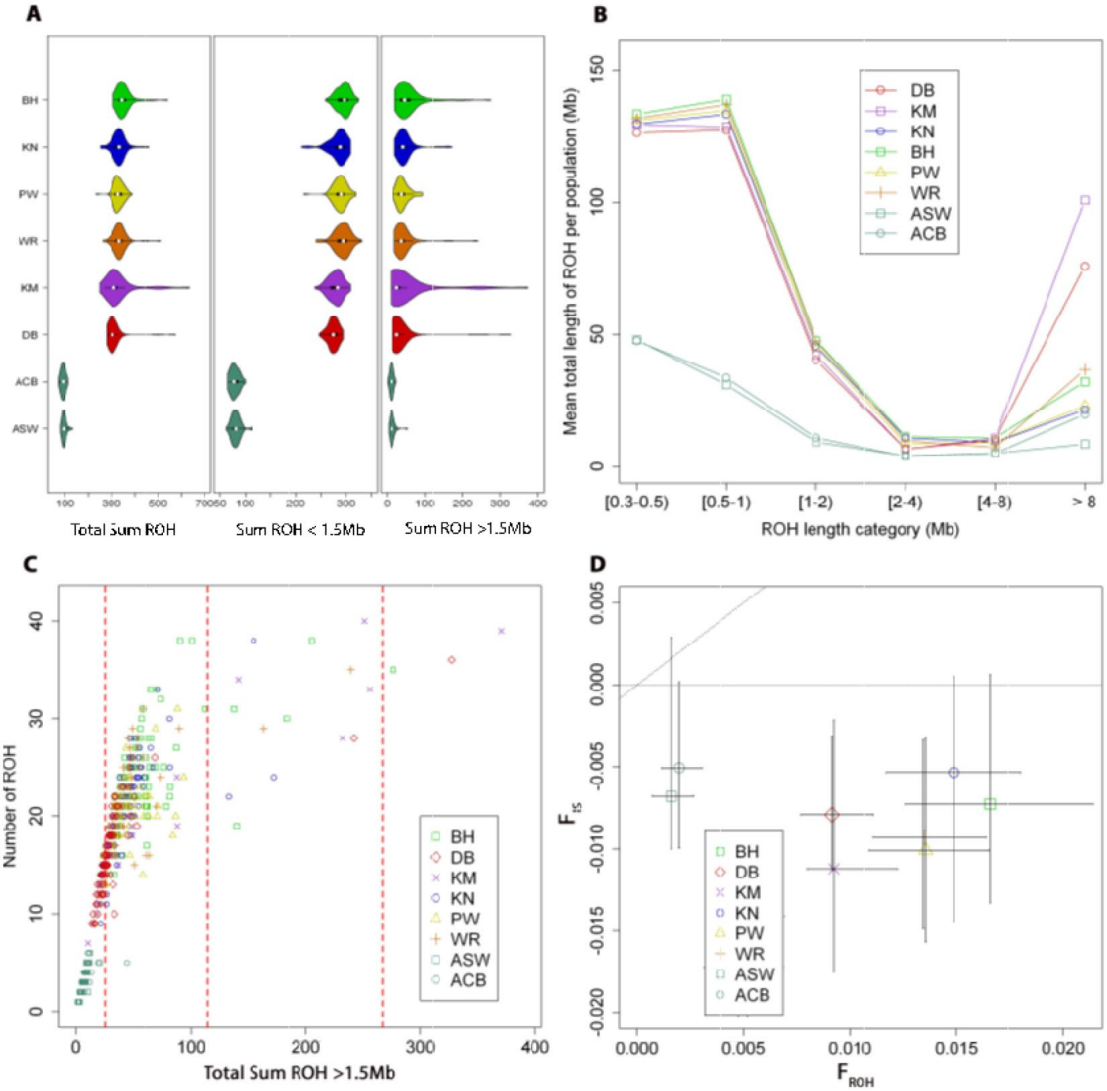
ROH analysis of the six West Maharashtrian populations. Deshastha Brahmins caste (DB) in red, Kunbi Marathas caste (KM) in violet, Kokana tribe (KN) in blue, Pawara tribe (PW) in yellow, Bhil tribe (BH) in green, and Warli tribe (WR) in orange. As admixed populations with no inbreeding we use ASW (Americans of African Ancestry in SW, USA) and ACB (African Caribbeans in Barbados) in turquoise from the 1KG cohort. (**A**) Violin plots showing the distribution of ROH within populations for the median total sum of ROH, median total sum of ROH shorter than 1.5 Mb and median total length of ROH longer than 1.5 Mb (white dots represent medians). (**B**) Mean total length of ROH (Mb) over six classes of ROH tract lengths. ROH classes: 0.3 < ROH < 0.5 Mb, 0.5 < ROH < 1 Mb, 1 < ROH < 2 Mb, 2<ROH < 4 Mb, 4<ROH < 8 Mb and ROH > 8 Mb. (**C**) Mean number of ROH and sum of ROH is plotted for each individual included in this study. The perpendicular broken red lines in at X = 25, X = 115 and X = 267 represent thresholds for second cousin, first cousin and uncle and niece offspring inbreeding coefficients. (**D**) Population Analysis and components of the inbreeding coefficient. The median of the systematic inbreeding (F_IS_) versus the median of the genomic inbreeding coefficient (F_ROH_) is shown for each population. Diagonal broken line represents F_IS_ = F_ROH_. Horizontal broken line represents F_IS_ = 0. IQR (Interquartile range) is shown for both F_IS_ and F_ROH_ in each population.

We first identified ROH in single individuals. The results are summarized in Fig. 1 and Supplementary Tables 1 and 2. The tribal groups have a slightly higher burden of total homozygosity, measured as the total sum of ROH (tribal average total sum of ROH = 0.34 Gb vs. caste average total sum of ROH = 0.31 Gb). Of the six WM groups, the Bhil and Warli tribes have the largest average total sum of ROH (0.35 and 0.34 Gb respectively, Supplementary Table 1) and also the largest average sum of short ROH (ROH < 1.5 Mb) (0.29 Gb). However, this is not the case for long ROH (ROH > 1.5 Mb), for which the Bhil tribe and the Kunbi Marathas caste show the highest average sum (0.058 and 0.052 Gb, respectively). It is interesting to note that for long ROH segments there are large differences between average and median values, particularly for the Bhil tribe and the Kunbi Marathas and Deshastha Brahmins castes (Fig. 1A). By classifying the mean total sum of ROH of each population in six ROH length classes (Fig. 1B) it is possible to see that the long tails seen in the sum of ROH > 1.5 Mb for both castes (Kunbi Marathas and Deshastha Brahmins) is due to a large burden of ROH > 8 Mb arising in the last 5 generations.

Population heterogeneity is better represented when the number of ROH and the total sum of ROH > 1.5 Mb are plotted for each individual (Fig. 1C). Vertical dashed red lines represent the cut-offs for individuals with F_ROH_ larger than a second cousin (F_ROH_ = 0.0152), first cousin (F_ROH_ = 0.0625) and uncle and niece (F_ROH_ = 0.125). The Kunbi Marathas sample has the highest proportion of individuals with genomic inbreeding coefficients (F_ROH_) higher than the offspring of both first cousin and uncle-niece mating (10.2% and 2% of the individuals sampled respectively, Supplementary Table 2). Even more, the two individuals with the highest F_ROH_ (0.1287 and 0.1137) belong to both castes (Kunbi Marathas and Deshastha Brahmins respectively) and can explain why they have a larger burden of ROH > 8 Mb. Besides these exceptions, Kunbi Marathas and Deshastha Brahmins are the populations with the lowest median F_ROH_ (0.0092 for both). In comparison with admixed populations, such as ACB and ASW, West Maharashtrian populations show, on average, F_RHO_ eight times larger. An important question to answer is whether this homozygosity is the result of consanguinity or isolation and genetic drift. In Fig. 1C it is possible to see that only a few Kunbi Maratha, Deshastha Brahmin, Bhil and Kokana individuals present a “shift-to-the-right” pattern when their number of ROH are plotted against their total sum of ROH > 1.5 Mb, as expected by consanguineous mating^34^. At a population level, this question is answered in Fig. 1D by plotting the population median F_IS_ against the population median FROH along with the interquartile range (IQR) of both variables. All the West Maharashtrian populations analysed are located under the F_IS_ = 0, which is consistent with a model based on isolation and genetic drift, and not systematic consanguinity.

### IBD analyses

Table 1 reports the average total length of the IBD segments (in centimorgans) shared per pair of individuals within and between groups. Within populations, the largest IBD sharing is observed in the Pawara and Bhil (69.47 cM per pair and 62.51 cM per pair, respectively), and the lowest IBD sharing in the two caste groups, Kunbi Marathas and Deshastha Brahmins (9.87 cM per pair and 2.82 cM per pair, respectively). Between populations, the largest IBD sharing is present between the Pawara and Bhil tribal groups (13.70 cM per pair) and the Kokana and Warli tribal groups (10.17 cM per pair). There is notably lower IBD sharing between individuals of the two caste groups (Kunbi Marathas and Deshastha Brahmins), as well as between the individuals of the two caste groups and the tribal groups. Supplementary Figures 1 and 2 show violin plots that depict the distribution of the total length of IBD segments shared between individuals within and between groups. Interestingly, although individuals from the Kunbi Marathas caste share a much lower proportion of their genome IBD than the individuals of any of the tribal groups (Table 1), the average size of the IBD segments shared by the Kunbi Marathas is the highest of all the groups analyzed here (6.26 cM, Table 2). In other words, although in the Kunbi Marathas we identified a much smaller number of IBD segments shared by individuals than in the tribal groups, the proportion of IBD segments of large size is higher (proportion of IBD segments larger than 15 cM: Kunbi Marathas: 8.85%, Pawara: 5.46%, Bhil: 3.47%, Kokana: 1.68%, Dehastha Brahmins: 1.02% and Warli: 0.74%).

**Table 1:** Average total length (in cM) of IBD segments shared per pair of individuals.

	BH	DB	KM	KN	PW	WR
BH	62.51					
DB	0.48	2.82				
KM	0.59	0.79	9.87			
KN	1.43	0.59	0.77	13.84		
PW	13.70	0.50	0.49	1.08	69.47	
WR	3.66	0.46	0.70	10.17	2.56	33.4

Diagonal: Within-populations, Below Diagonal: Between-populations.

**Table 2:** Average size of the shared IBD segments (in cM).

	BH	DB	KM	KN	PW	WR
BH	5.05					
DB	2.53	3.54				
KM	2.61	2.61	6.26			
KN	2.69	2.64	2.71	4.21		
PW	3.55	2.52	2.57	2.62	5.77	
WR	2.74	2.55	2.67	3.09	2.71	3.51

Diagonal: Within-populations, Below Diagonal: Between populations.

PCA analysis of modern and ancient South Asian samples.

We generated a network of IBD sharing between individuals based on the full IBD dataset, and also on segments of different sizes (Supplementary Figure 3). The lengths of IBD segments will depend on the number of generations separating the two haplotypes that share a segment from their common ancestor^35^. Individuals that share higher proportions of their genome IBD are located closer in the graphs. In the plots based on the full IBD dataset, the four tribal groups are clearly separated from the two caste groups. In agreement with the results based on the average total length of IBD shared between pairs of individuals, members of the Pawara tribal group clearly cluster with members of the Bhil tribal group, and individuals from the Kokana tribal group are located close to those of the Warli tribal group. Further, the plots based on large IBD segments (e.g. ≥14 cM) highlight that individuals from the tribal groups share more recent common ancestry than the individuals of the two caste groups. Particularly, the shared recent common ancestry between individuals from the Pawara and Bhil tribes is clear in the figures.

We also estimated effective population sizes for the six WM groups (Supplementary Figure 4). In general, effective population sizes have been lower in the tribal population groups than in the castes. Among the tribal groups, the Warli shows a larger increase in population size within the last 20 generations than the other three groups. The patterns observed for the two caste groups are quite different. In the Deshastha Brahmins group, there has been a relatively constant population size and then a major population expansion within the last ten generations. In the Kunbi Marathas, the most notable aspect of the plot is the sharp drop in effective population size that started approximately 15 generations ago, with a recent increase about six generations ago.

### PCA analysis of modern and ancient South Asian samples

Supplementary Figure 5 depicts the PCA plot of the WM samples with modern South Asian samples. The samples are distributed in a cline in the 1^st^ PC, with the Punjabi samples occupying the most positive regions and several Dravidian tribal and caste groups from the SGDP project (Irula, Madiga and Mala) located on negative regions in the 1^st^ PC. The two WM caste samples overlap with the 1KG samples. The four WM tribal groups are clearly separated from the two caste samples and all the 1KG and SGDP samples, occupying the most negative regions of the 1^st^ PC. Additionally, there is a clear split between the tribal samples in the 2^nd^ PC, with the Pawara and Bhil clustering together in positive regions and the Warli and Kokana in negative regions. As an alternative strategy to depict population relationships, Supplementary Figure 6 shows a Neighbor-Joining tree including the WM samples with the 1KG South Asian samples, using an African sample as an outgroup. The tree was based on FST distances. The dendrogram is very consistent with the PC plots, with the four WM tribal groups clustering apart from all the caste groups.

**Figure 2:**
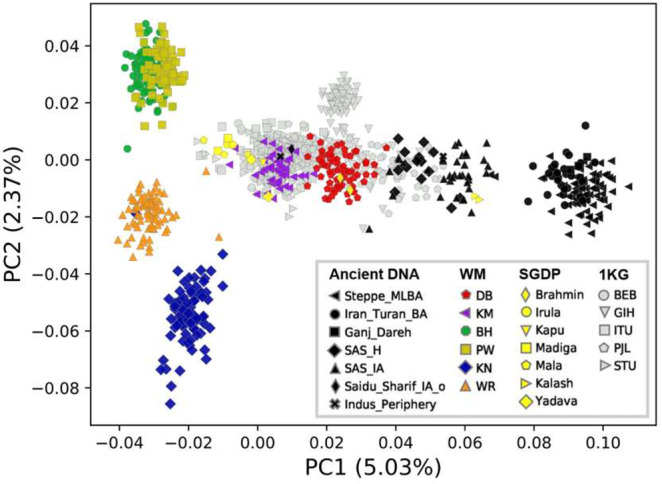
PCA plot including the tribal and caste groups from West Maharashtra, as well as relevant modern and ancient DNA samples. The ancient DNA samples were projected onto the modern DNA samples.

We also carried out a PCA analysis projecting ancient DNA samples onto the modern South Asian samples (Fig. 2). The ancient DNA sample includes early goat herders from Zagros Mountains of Iran (Ganj-Dareh); several Bronze Age samples from Iran_Turan (Iran-Turan BA); three Bronze Age samples from Iran-Turan (Shahr_I_Sokhta_BA2, Shahr_I_Sokhta_BA3 and Gonur2) that Narasimhan *et al*.^5^ referred to as “Indus Periphery” samples and show a complex mixture of ancestry related to Iranian agriculturalists and South Asian hunter-gatherers (but no Steppe ancestry); Middle-to-late Bronze age samples from the Steppe (Steppe-MLBA); South Asian samples from the Iron Age and the Holocene (SAS_IA and SAS_H, respectively); and an Iron Age outlier from the South Asian Iron Age sample without Steppe ancestry (Saidu_Sharif_IA_o). A cline that is consistent with the well-known ANI-ASI cline is observed in the 1^st^ PC. The most negative regions of this axis are occupied by the WM tribal groups and also modern samples from South India, most of them Dravidian-speakers (Madiga, Mala, Irula and some 1KG Sri Lankan Tamil and Telugu samples). The Steppe-MLBA samples and the Ganj-Dareh samples (and some Iran_Turan_BA samples) occupy the most positive regions of the 1^st^ PC. The SAS_H samples tend to show higher ASI ancestry than the earlier SAS_IA samples. The Indus_Periphery samples are located within the region occupied by the modern Indian populations, but they do not cluster together in the plot. We also prepared a PCA plot without projecting the ancient DNA samples (Supplementary Figure 7). In this plot, the Steppe-MLBA samples and the Ganj-Dareh samples are separated in the 2^nd^ PC.

### Unsupervised clustering analysis of modern and ancient South Asian DNA samples

**Figure 3:**
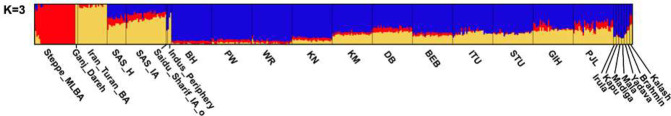
Unsupervised clustering analysis using the program ADMIXTURE using K = 3, including tribal and caste groups from West Maharashtra, as well as relevant modern and ancient DNA samples.

To infer in more detail individual clustering and potential admixture patterns in the modern and ancient DNA samples, we used an unsupervised ADMIXTURE analysis at K = 3 (Fig. 3). This model was chosen given that in their comprehensive analysis of modern South Asian samples and ancient DNA samples from South and Central Asia, Narasimhan *et al*.^5^ have shown that modern South Asian populations are primarily the result of admixture between three distinct population groups: AASI, Ancient Iranians, and Steppe groups. The results for the samples presented in Narasimhan *et al*.^5^ are fully concordant with the results described in their study. Steppe_MBLA samples primarily fall into a red component, whereas the samples from Ganj-Dareh in Iran form a different yellow component. There is also a blue component that appears in different proportions in many of the modern and ancient samples, which seems to represent the AASI contribution. Most of the samples in the analysis (except Steppe-MLBA and Ganj_Dareh) appear to be the result of admixture between these ancestral groups (Steppe, ancient Iran and AASI). The average contributions of each component to each sample are depicted in Supplementary Table 3. In the modern samples, the highest Steppe contributions (>10%) are found in the 1KG Punjabi, the WM Dehastha Brahmin and the SGDP Brahmin. Very high AASI contributions are observed in the WM tribal samples, in particular the Bhil (91.5%), Warli (91.4%) and Pawara (88.5%). We also carried out an unsupervised ADMIXTURE analysis using Andamanese samples (Onge and Jarawa, Supplementary Figure 8), with very similar results. In these analyses, the Andamanese samples have a different component to those determined by the Steppe groups, ancient Iranians and the putative AASI component.

### Allele sharing analyses and admixture models

We carried out analyses using the *f3*- and *f4*-statistics to further explore the relationships of the WM tribal and caste samples, as well as 1KG and SGDP modern samples from South Asia, with relevant ancient DNA samples (Supplementary Figures 9–14 and Supplementary Tables 4–9). The plots are concordant with the PCA and ADMIXTURE results described above. In the outgroup *f3* plots, the highest values of shared genetic drift with the Andamanese sample are observed for the WM tribal groups, and also other tribal groups from South Asia, such as Mala and Madiga (Supplementary Figure 9). The lowest shared genetic drift is observed with the samples from Pakistan (SGDP Kalash and 1KG Punjabi), and in India with the SGDP Brahmin sample, the WM Deshastha Brahmin sample and the 1KG Gujarati sample. The opposite patterns are observed when exploring shared drift with the ancient Iranians (Supplementary Figure 10) and the Steppe samples (Supplementary Figure 11). The *f4*-statistics highlight very similar patterns, but provide additional information (Supplementary Figures S12-S14). The tribal groups from WM, as well as some Dravidian tribal and caste groups (Mala, Madiga and Irula) share less alleles with Ganj-Dareh and Steppe-MLBA samples than other non-tribal groups. In particular, Kalash and Punjabis from Pakistan and Brahmin and Gujarati from India share more alleles with the Steppe pastoralist and ancient Iranian groups than other Indian non-tribal groups, such as the WM Kunbi Marathas, Bengali from Bangladesh, Sri Lankan Tamil and Indian Telugu.

To estimate the relative contributions of AASI-related (Andamanese), Indus-Diaspora-related and Steppe-related groups to the WM tribal and caste populations, as well as other relevant groups from South Asia we used the program qpAdm. Supplementary Table 10 provides the estimates of the relative admixture proportions from each source group for each target population. The p-values are higher than 0.01 for all of the target populations, except Deshastha Brahmin (p = 0.008), indicating a good fit of the models. The highest AASI-related contribution was observed for the Warli tribal group (0.54) and the highest Steppe-related contributions in Punjabi (0.191). Of note, in agreement with the ADMIXTURE estimates, the Steppe contributions in the tribal Indo-European groups from WM are higher than in Dravidian tribes and castes (Mala, Irula, Telugu, Kapu and Madiga). The highest contributions from Indus-Diaspora-related groups are in Telugu, SGDP Brahmin and Gujarati.

## Discussion

Genetic studies in South Asia, including evidence from mtDNA^36–46^ and Y-chromosome^41,47–54^ markers and dense autosomal datasets in modern^4,8,55–58^ and ancient DNA samples^59,60^ have provided fascinating insights about the demographic history of the region. These studies have explored a wide range of topics, including the genetic relationships between populations belonging to distinct linguistic groups (e.g. Indo-European, Dravidian, Austro-Asiatic and Tibeto-Burman), the genetic diversity present in castes and tribal groups, as well as the history and pattern of migrations in this region. The recent availability of ancient DNA data has added an important temporal perspective, making it possible to study in much more detail the relative contributions of Ancient Ancestral South Indians (AASI), Ancient Iranians and Steppe populations to modern tribal and caste groups across South Asia^5–7^.

In this study we generated genome-wide data from two castes (Deshastha Brahmin and Kunbi Maratha) and four tribal groups (Kokana, Warli, Bhil and Pawara) from the Indian state of West Maharashtra, and investigated the demographic history of this region in addition to the genetic relationships of these populations with other modern and ancient samples from the South Asian continent.

We identified ROH in single individuals and IBD segments in pairs of individuals within and between groups. The homozygosity patterns observed in the six WM groups is primarily the result of isolation and genetic drift, rather than systematic inbreeding (Fig. 1). Within-population IBD analyses show that individuals within the tribal groups share a much larger proportion of their genome IBD than individuals within the two castes (Table 1, Supplementary Figure 1). Between-population IBD analyses point to shared recent common ancestry between individuals of the Bhil and Pawara tribal groups, and to a lesser extent between individuals of the Warli and Kokana tribal groups (Table 1, Supplementary Figures 2 and 3). In contrast, all the other pairwise population comparisons indicate much more limited IBD sharing. The Bhil and Pawara live in the same geographic area within the Nandurbar district in northern Maharashtra. The Pawara are often considered a sub-division within the Bhil groups^61^ but these are identified as different tribal groups by Enthoven^62^. The Kokana and Warli groups also live in the same geographic region in the Palghar district. Close affinity between Bhil and Pawara and to a lesser degree between Kokana and Warli was also previously shown by dental nonmetric traits^63^ and mitochondrial D-loop sequences^64^.

We observed clear differences in effective population sizes between the WM tribal and caste groups (Supplementary Figure 4). The four tribal populations show substantially smaller effective population sizes than the caste groups. Furthermore, in three of the four groups (Bhil, Pawara and Kokana) there is a decrease in effective population size starting less than 20 generations ago, prior to a rebound in effective size that is evident starting approximately ten generations ago. This decline in effective population size is not observed in the Warli tribal group. The effective sizes of the two castes are larger than the tribal groups, but their demographic patterns are quite different. In particular, in the Kunbi Marathas, there is evidence of quite a dramatic effective population drop starting 20 generations ago (Supplementary Figure 4). This is consistent with our observation that the Kunbi Marathas sample is characterized by a relative enrichment in long ROH and IBD segments with respect to other groups. Although it is difficult to pinpoint the possible causes for the population declines of the groups analyzed here, particularly within the Kunbi Marathas, there have been several mega-famines in India as a result of multi-year failures of the monsoon, including the devastating Durga Devi famine that lasted more than a decade, from 1396 AD to 1409 AD^65,66^.

In order to gain further insights on the relationships of the WM caste and tribal groups with other South Asian populations, we carried out a number of analyses that included other modern South Asian groups (SGDP and 1KG South Asian groups), as well as ancient DNA samples from this region^5,6^. The analyses using PCA, ADMIXTURE, *f3* and *f4* statistics, and qpAdm show very consistent patterns. In the PCA plots, the tribal groups from WM split from other tribal and caste South Asian groups in the second PC (Fig. 1 and Supplementary Figure 5). Given the small effective population sizes of the tribes, this observation is likely due to the influence of genetic drift (Supplementary Figure 4). Also, in agreement with the IBD analyses (Supplementary Figure 3), individuals from the Bhil and Pawara tribal groups are located closer together in the PCA plot indicating strong genetic affinities, and individuals from the Kokana and Warli tribes are seen to separate from other populations (Fig. 2 and Supplementary Figure 5).

The availability of ancient DNA data for Steppe pastoralists, Ancient Iranians and individuals from the Indus-Periphery makes it possible to obtain much more nuanced insights on the relationships of the WM tribal and caste groups with other populations from India and Pakistan, and also on the fit of the WM populations to the well-known South Asian cline. In this respect, it is important to highlight the differences in the type of information provided by the program ADMIXTURE and *f3*, *f4* and qpAdm statistics. The program ADMIXTURE provides estimates of relative ancestral contributions using an unsupervised clustering strategy based on modern and ancient samples. The results obtained using a model with three parental groups identifies two ancestral components present in modern South Asian populations that are maximized in the Steppe pastoralists (Steppe_MLBA) and Ancient Iranians (Ganj_Dareh), and a third component that is also present in many ancient and modern South Asian populations, and is particularly prevalent in the WM tribal groups and in Dravidian tribes and castes. This third component seems to represent the AASI contributions, and our results for the ancient DNA samples and modern SGDP and 1KG samples are in broad agreement with those reported by Narashiman *et al*.^5,6^. Unfortunately, there are no unadmixed modern or ancient representatives of the AASI population. In contrast, the analyses using *f3*- and *f4*-statistics and qpAdm are driven by comparisons with reference samples. For the analyses with *f3*- and *f4*-statistics, we used the Andamanese as a population distantly related to the AASI group, and the Ganj_Dareh and Steppe_MLBA samples as representative of the Ancient Iranians and Steppe pastoralists, respectively. For the analyses with qpAdm we used the same source populations and outgroups as those used by Narasimhan *et al*.^5,6^. Using this admixture model we can compare the results obtained for the WM samples with the extensive dataset of approximately 140 South Asian groups presented in Narasimhan *et al*.^5,6^. When interpreting the qpAdm results, it is important to note that the Onge population is only very distantly related to the AASI population and that the Indus-Periphery samples have both Ancient Iranian and AASI contributions. In spite of these caveats, all our analyses indicate that the WM tribal groups have high AASI contributions, similarly to tribal and caste Dravidian populations (Irula, Mala, Madiga and Kapu). This is evident in the large putative AASI components in the ADMIXTURE plots (Fig. 3), and the high Onge (AASI)-related contribution observed in the qpAdm analyses (Supplementary Table 10).

Our results are also consistent with the qpAdm results reported by Narasimhan *et al*.^5^, which included small Bhil (N = 8) and Warli (N = 3) samples. In their analyses, the Warli had one of the highest Onge (AASI)-related contributions reported for the Indian cline groups, as well as the Kolcha (another Indo-european tribe) and Dravidian populations from South India such as the Kurchas, Irula, Malayan, Adiyan, Ulladan, Palliyar and Pulliyar. Although both Indo-European and Dravidian tribal groups have very high AASI contributions, Indo-European tribal groups tend to have higher Steppe contributions than Dravidian tribal groups. This provides indirect support for a model that explains the spread of the Indo-European languages as a result of the migration of the Steppe pastoralists to Europe and South Asia, in contrast to the alternative model that supports the spread of Indo-European languages as a consequence of the migration of Anatolian farmers. This Steppe hypothesis has also been recently supported by Shinde *et al*.^7^, who have shown that there is negligible ancestry from Anatolian farmers in the ancient South Asian farmers represented in the Indus Valley Civilization. Overall, our results are in agreement with recent ancient DNA studies providing support for Steppe pastoralists as a source of Indo-European languages in Europe and South Asia^6,7,67^. Comparisons of mtDNA, Y-chromosome and autosomal data indicate that this migration was primarily male-driven^40^.

In summary, in this study we present novel insights on the demographic history and population relationships of six Indo-European populations from the Indian state of West Maharashtra (four tribal groups and two castes). We show that WM tribal groups have smaller effective population sizes than WM castes, and genetic drift has had a higher impact in tribal populations. We also show clear affinities between the Bhil and Pawara tribes, and to a lesser extent, between the Warli and Kokana tribes. Our comparisons with available genomic data from modern and ancient samples from South Asia indicate higher Ancient Iranian and Steppe pastoralist contributions in the WM Brahmin caste than in the WM Kunbi Marathas caste. In contrast to the two castes, WM tribal groups have very high AASI contributions. Although both Indo-European and Dravidian tribal groups from India have very high AASI contributions, Indo-European tribal groups tend to have higher Steppe contributions than Dravidian tribal groups, providing further support for the hypothesis that Steppe pastoralists were the source of Indo-European languages in South Asia.

## Supplementary information

Supplementary information.

## Data Availability

The dataset analysed and presented during the current study are available from the corresponding author on reasonable request.

## References

[1] Malhotra, K. C. Population Structure among the Dhangar Caste-Cluster of Maharashtra, India. In The People of South Asia 295–324, 10.1007/978-1-4899-5001-7_14(Springer US, 1984).

[2] India, G. of. Statistical Profile of Scheduled Tribes in India. Ministry of Tribal Affairs, Statistics Division, Government of India. (2013).

[3] Reddy BM, Tripathy V, Kumar V, Alla N (2010). Molecular genetic perspectives on the Indian social structure. Am. J. Hum. Biol..

[4] Reich D, Thangaraj K, Patterson N, Price AL, Singh L (2009). Reconstructing Indian population history. Nature.

[5] Narasimhan, V. M. *et al*. The genomic formation of South and Central Asia. bioRxiv 292581 (2018).

[6] Narasimhan, V. M. *et al*. The formation of human populations in South and Central Asia. *Science* **365**, eaat7487 (2019). 10.1126/science.aat7487 PMC6822619 31488661

[7] Shinde V (2019). An Ancient Harappan Genome Lacks Ancestry from Steppe Pastoralists or Iranian Farmers. Cell.

[8] Moorjani P (2013). Genetic evidence for recent population mixture in India. Am. J. Hum. Genet..

[9] Jonnalagadda M, Ozarkar S, Ashma R, Kulkarni S (2016). Skin pigmentation variation among populations of West Maharashtra. India. Am. J. Hum. Biol..

[10] Sambrook, J. & Russell, D. W. Molecular Cloning. (CSHL Press, 2001).

[11] Delaneau O, Marchini J, Zagury J-F (2012). A linear complexity phasing method for thousands of genomes. Nat. Methods.

[12] Purcell S (2007). PLINK: a tool set for whole-genome association and population-based linkage analyses. Am. J. Hum. Genet..

[13] Ceballos FC, Hazelhurst S, Ramsay M (2018). Assessing runs of Homozygosity: A comparison of SNP Array and whole genome sequence low coverage data. BMC Genomics.

[14] Ceballos FC, Hazelhurst S, Ramsay M (2019). Runs of homozygosity in sub-Saharan African populations provide insights into complex demographic histories. Hum. Genet..

[15] International HC (2007). A second generation human haplotype map of over 3.1 million SNPs. Nature.

[16] Slatkin M (2008). Linkage disequilibrium - Understanding the evolutionary past and mapping the medical future. Nat. Rev. Genet..

[17] Malécot G (1969). Consanguinité panmictique et consanguinité systématique. Ann. Génétique Sélection Anim..

[18] Templeton AR, Read B (1994). Inbreeding: one word, several meanings, much confusion. EXS.

[19] Hartl, D. L. & Clark, A. G. Principles of Population Genetics. (Sinauer Associates Incorporated, 2007).

[20] Nei M (1977). F-statistics and analysis of gene diversity in subdivided populations. Ann. Hum. Genet..

[21] Weir BS (2012). Estimating F-statistics: A historical view. Philos. Sci..

[22] McQuillan R (2008). Runs of Homozygosity in European Populations. Am. J. Hum. Genet..

[23] Browning BL, Browning SR (2013). Improving the accuracy and efficiency of identity-by-descent detection in population data. Genetics.

[24] Bastian, M., Heymann, S. & Jacomy, M. Gephi: an open source software for exploring and manipulating networks. In vol. Third inte (2009).

[25] Browning SR, Browning BL (2015). Accurate non-parametric estimation of recent effective population size from segments of identity by descent. Am. J. Hum. Genet..

[26] Auton A (2015). A global reference for human genetic variation. Nature.

[27] Mallick S (2016). The Simons Genome Diversity Project: 300 genomes from 142 diverse populations. Nature.

[28] Patterson N, Price AL, Reich D (2006). Population structure and eigenanalysis. PLoS Genet..

[29] Price AL (2006). Principal components analysis corrects for stratification in genome-wide association studies. Nat. Genet..

[30] Alexander DH, Novembre J, Lange K (2009). Fast model-based estimation of ancestry in unrelated individuals. Genome Res..

[31] Petr M, Vernot B, Kelso J (2019). admixr—R package for reproducible analyses using ADMIXTOOLS. Bioinformatics.

[32] Patterson N (2012). Ancient admixture in human history. Genetics.

[33] Lazaridis I (2016). Genomic insights into the origin of farming in the ancient Near East. Nature.

[34] Ceballos FC, Hazelhurst S, Ramsay M (2018). Assessing runs of Homozygosity: a comparison of SNP Array and whole genome sequence low coverage data. BMC Genomics.

[35] Browning SR (2008). Estimation of pairwise identity by descent from dense genetic marker data in a population sample of haplotypes. Genetics.

[36] Atkinson QD, Gray RD, Drummond A (2008). J. mtDNA Variation Predicts Population Size in Humans and Reveals a Major Southern Asian Chapter in Human Prehistory. Mol. Biol. Evol..

[37] Chandrasekar A (2009). Updating Phylogeny of Mitochondrial DNA Macrohaplogroup M in India: Dispersal of Modern Human in South Asian Corridor. PLoS ONE.

[38] Palanichamy M (2004). Phylogeny of mitochondrial DNA macrohaplogroup N in India, based on complete sequencing: implications for the peopling of South Asia. Am. J. Hum. Genet..

[39] Sun C (2006). The Dazzling Array of Basal Branches in the mtDNA Macrohaplogroup M from India as Inferred from Complete Genomes. Mol. Biol. Evol..

[40] Metspalu M (2004). Most of the extant mtDNA boundaries in South and Southwest Asia were likely shaped during the initial settlement of Eurasia by anatomically modern humans. BMC Genet..

[41] Kivisild T (2003). The Genetic Heritage of the Earliest Settlers Persists Both in Indian Tribal and Caste Populations. Am. J. Hum. Genet..

[42] Mellars P, Gori KC, Carr M, Soares PA, Richards MB (2013). Genetic and archaeological perspectives on the initial modern human colonization of southern Asia. Proc. Natl. Acad. Sci..

[43] Silva M (2017). A genetic chronology for the Indian Subcontinent points to heavily sex-biased dispersals. BMC Evol. Biol..

[44] Thangaraj K (2003). Genetic Affinities of the Andaman Islanders, a Vanishing Human Population. Curr. Biol..

[45] Thangaraj K (2010). The Influence of Natural Barriers in Shaping the Genetic Structure of Maharashtra Populations. PLoS ONE.

[46] Chaubey G (2008). Phylogeography of mtDNA haplogroup R7 in the Indian peninsula. BMC Evol. Biol..

[47] Bamshad M (2001). Genetic Evidence on the Origins of Indian Caste Populations. Genome Res..

[48] Cordaux R (2004). Independent Origins of Indian Caste and Tribal Paternal Lineages. Curr. Biol..

[49] Ramana GV (2001). Y-chromosome SNP haplotypes suggest evidence of gene flow among caste, tribe, and the migrant Siddi populations of Andhra Pradesh, South India. Eur. J. Hum. Genet..

[50] Sahoo S (2006). A prehistory of Indian Y chromosomes: Evaluating demic diffusion scenarios. Proc. Natl. Acad. Sci. U. S. A..

[51] Sengupta S (2006). Polarity and Temporality of High-Resolution Y-Chromosome Distributions in India Identify Both Indigenous and Exogenous Expansions and Reveal Minor Genetic Influence of Central Asian Pastoralists. Am. J. Hum. Genet..

[52] Zerjal T (2006). Y-chromosomal insights into the genetic impact of the caste system in India. Hum. Genet..

[53] Borkar M (2011). Paleolithic spread of Y-chromosomal lineage of tribes in eastern and northeastern India. Ann. Hum. Biol..

[54] Khan F (2007). Genetic affinities between endogamous and inbreeding populations of Uttar Pradesh. BMC Genet..

[55] Metspalu M (2011). Shared and unique components of human population structure and genome-wide signals of positive selection in South Asia. Am. J. Hum. Genet..

[56] Majumder PP, Basu A (2014). A genomic view of the peopling and population structure of India. Cold Spring Harb. Perspect. Biol..

[57] Basu A, Sarkar-Roy N, Majumder PP (2016). Genomic reconstruction of the history of extant populations of India reveals five distinct ancestral components and a complex structure. Proc. Natl Acad. Sci. USA.

[58] Mondal M (2016). Genomic analysis of Andamanese provides insights into ancient human migration into Asia and adaptation. Nat. Genet..

[59] Narasimhan VM (2019). The formation of human populations in South and Central Asia. Science.

[60] Shinde V (2019). An Ancient Harappan Genome Lacks Ancestry from Steppe Pastoralists or Iranian Farmers. Cell.

[61] Bhanu, B. V. *People of India*. (Popular Prakashan, 2004).

[62] Enthoven, R. E. *The Tribes and Castes of Bombay*. (Asian Educational Services, 1990).

[63] Jonnalagadda M, Nagare T, Chitale S (2013). A. O. Population Affinities of Select Tribal Populations of Maharashtra: A Study Using Dental Morphology. Ind J Phys Anthr. Hum. Genet..

[64] Ozarkar, S. Mitochondrial DNA Diversity among Select Tribal Populations of Maharashtra: PhD Thesis submitted to Savitribai Phule Pune University.

[65] Sinha, A. et al. A 900-year (600 to 1500 A.D.) record of the Indian summer monsoon precipitation from the core monsoon zone of India. Geophys. Res. Lett. 34 (2007).

[66] Wilson IRG (2009). Can we Predict the Next Indian Mega-Famine. Energy Environ..

[67] Haak W (2015). Massive migration from the steppe was a source for Indo-European languages in Europe. Nature.

