# Supplementary material for: Novel insights on demographic history of tribal and caste groups from West Maharashtra (India) using genome-wide data
Source: Sci Rep. 2020 Jun 22;10:10075. doi: 10.1038/s41598-020-66953-3 (PMC7308293; doi:10.1038/s41598-020-66953-3)
Supplement: Supplementary file 1 — Supplementary information. [file 41598_2020_66953_MOESM1_ESM.pdf]

Novel insights on demographic history of tribal and caste groups from West Maharashtra (India) using genome-wide data.

Guilherme Debortoli[1†], Cristina Abbatangelo[1†], Francisco Ceballos[2†], Cesar Fortes-Lima[3], Heather L. Norton[4], Shantanu Ozarkar[5], Esteban J. Parra[1], Manjari Jonnalagadda[6*].

1. *Department of Anthropology, University of Toronto at Mississauga, Mississauga, ON, Canada*
2. *Department of Biological Sciences, Middle East Technical University, Çankaya, Turkey*
3. *Sub-department of Human Evolution, Department of Organismal Biology, Evolutionary Biology Centre, Uppsala University, Uppsala, Sweden*
4. *Department of Anthropology, University of Cincinnati, Cincinnati, OH, USA*
5. *Department of Anthropology, Savitribai Phule Pune University, India*
6. *Symbiosis School for Liberal Arts, Symbiosis International (Deemed University), Pune, India*

[†]*These authors equally contributed to the publication*

[*]*Corresponding author*

Manjari Jonnalagadda, Ph.D.

Symbiosis School for Liberal Arts, Symbiosis International (Deemed University), Pune, India.
E-mail: manjari.jonnalagadda@ssla.edu.in

Supplementary Figure 1: Violin plot of sum of IBD segments (in cM) shared between individuals from each group.

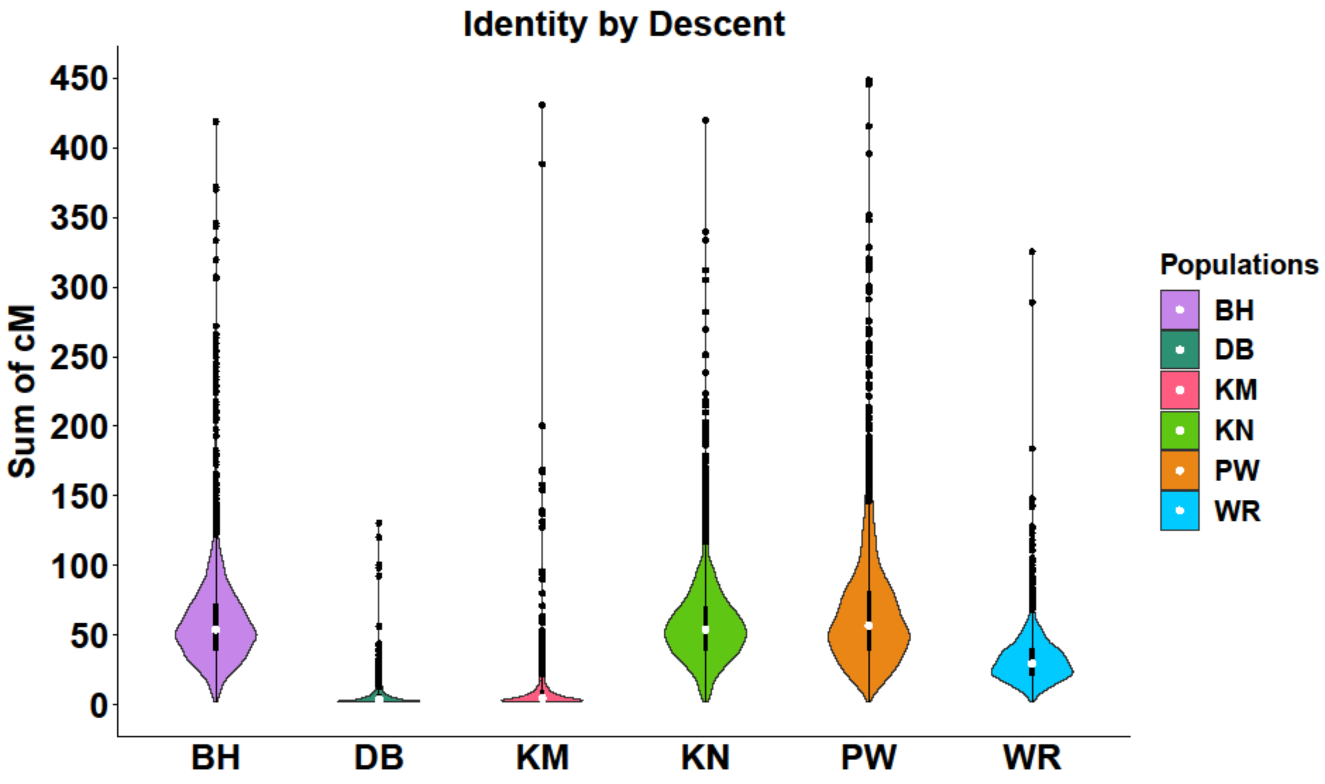

Supplementary Figure 2: Violin plot of sum of IBD segments (in cM) shared between individuals from different groups.

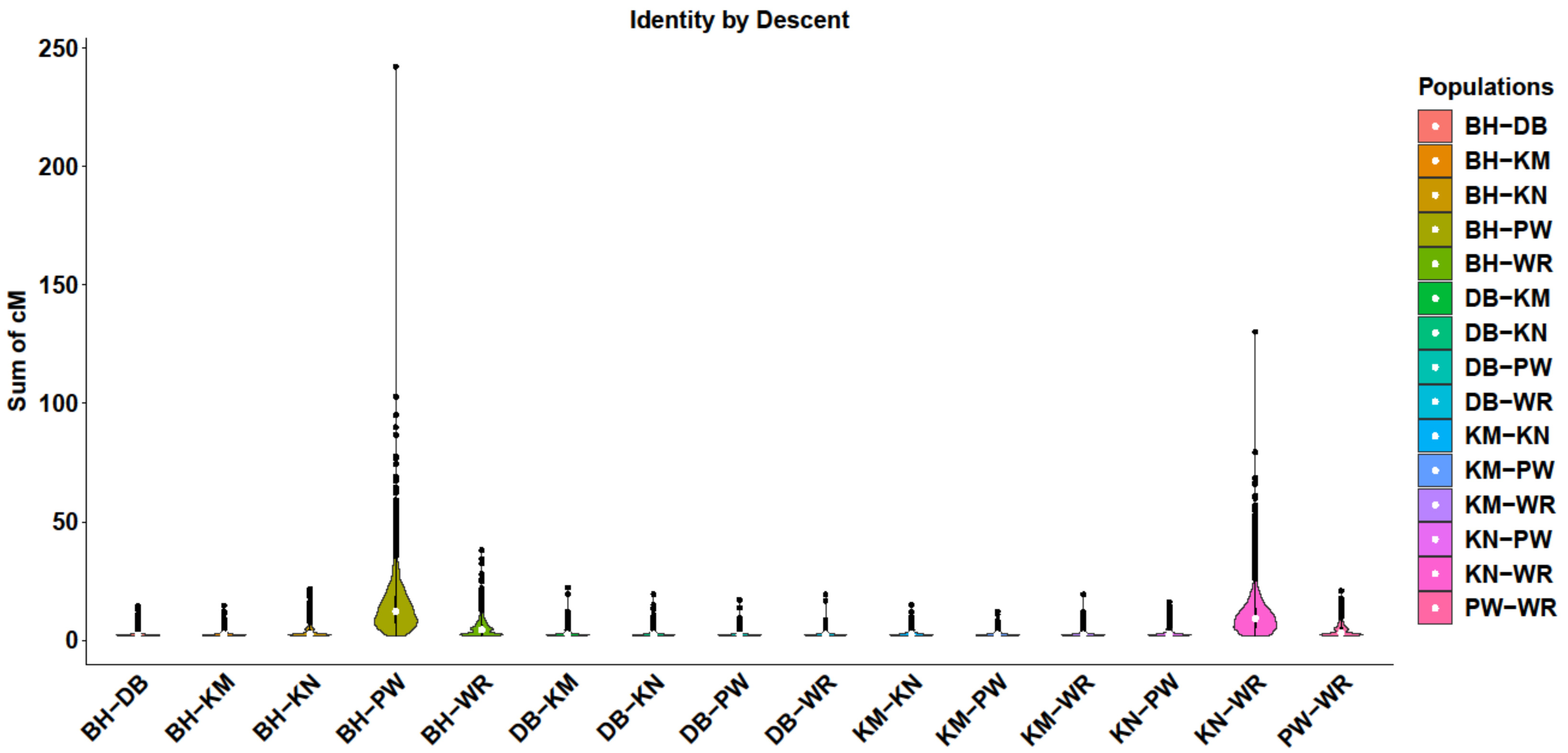

Supplementary Figure 3: We used the program Gephi (version 0.9.2)[1] to plot IBD sharing between individuals based on the full IBD dataset, and also on segments of different sizes. Individuals that share higher proportions of their genome IBD are located closer in the graphs.

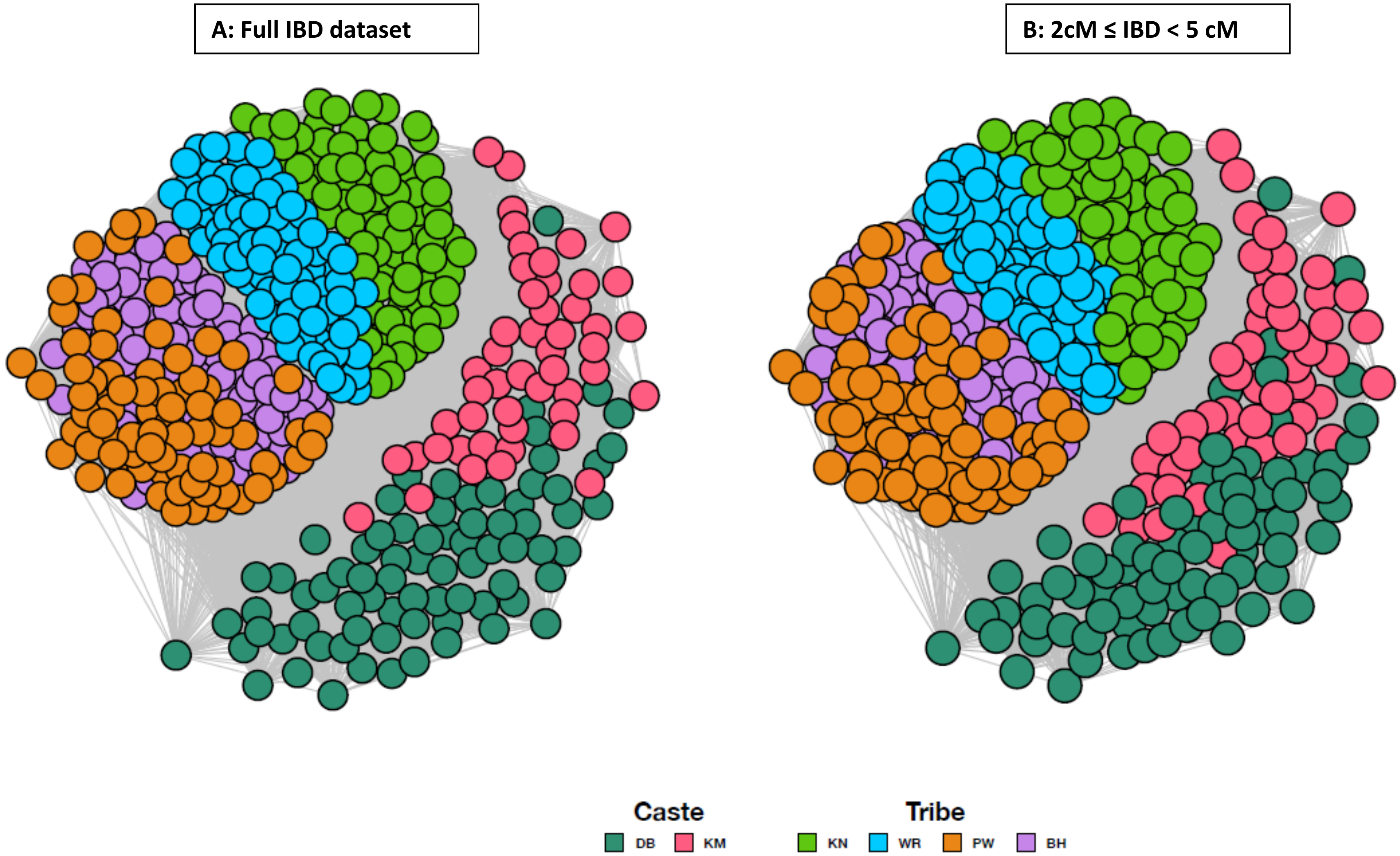

Supplementary Figure 3 (continuation)

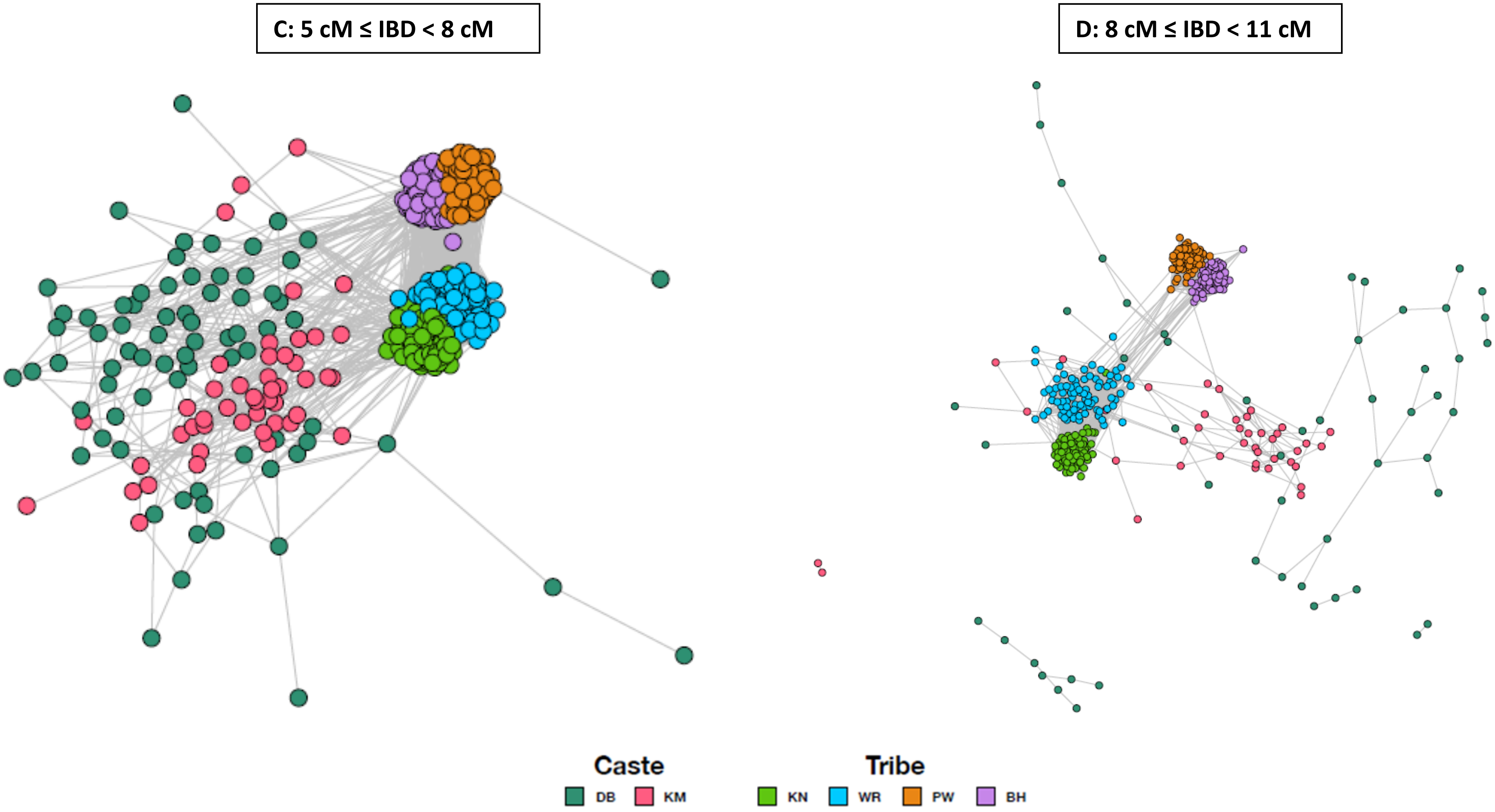

Supplementary Figure 3 (continuation)

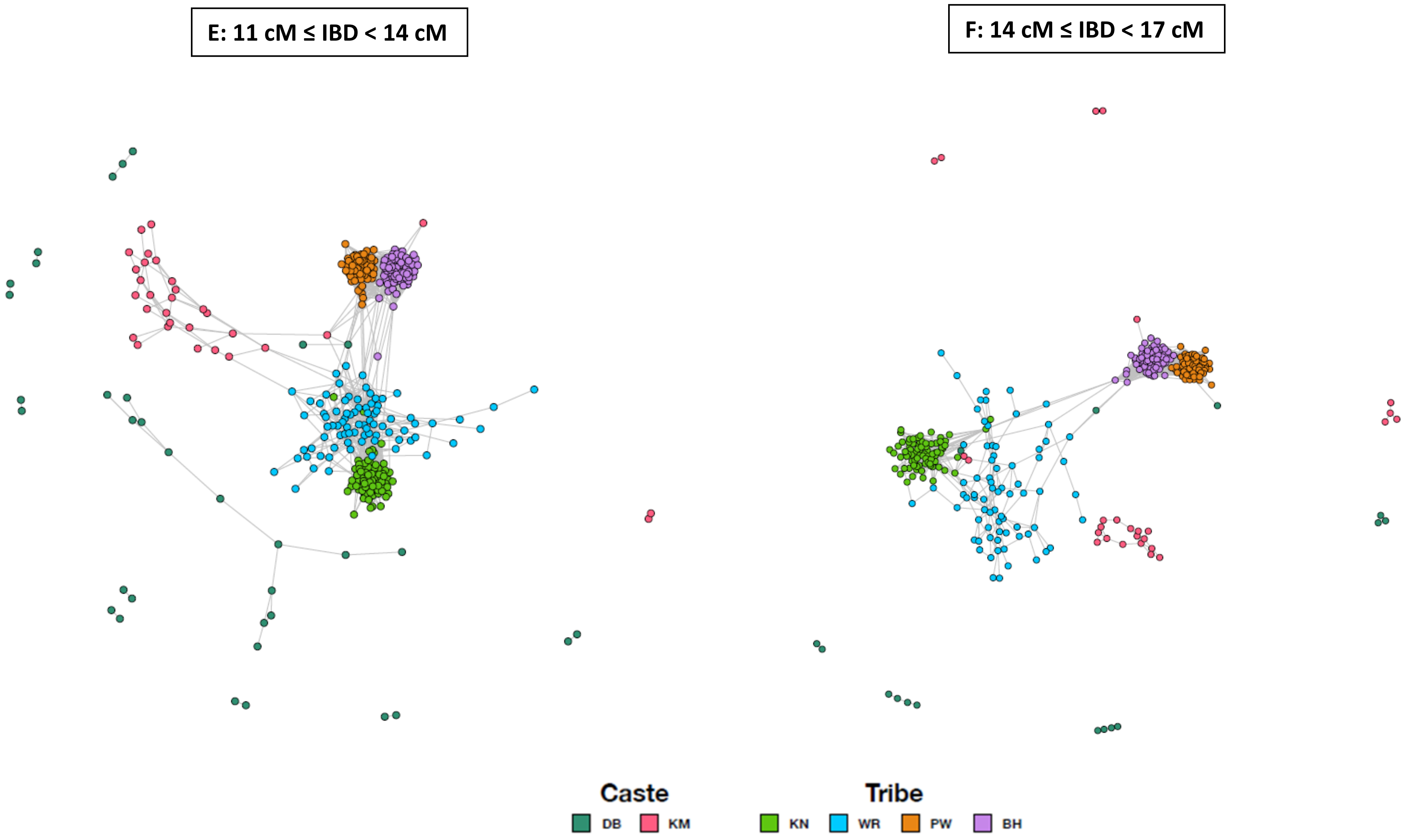

Supplementary Figure 3 (continuation)

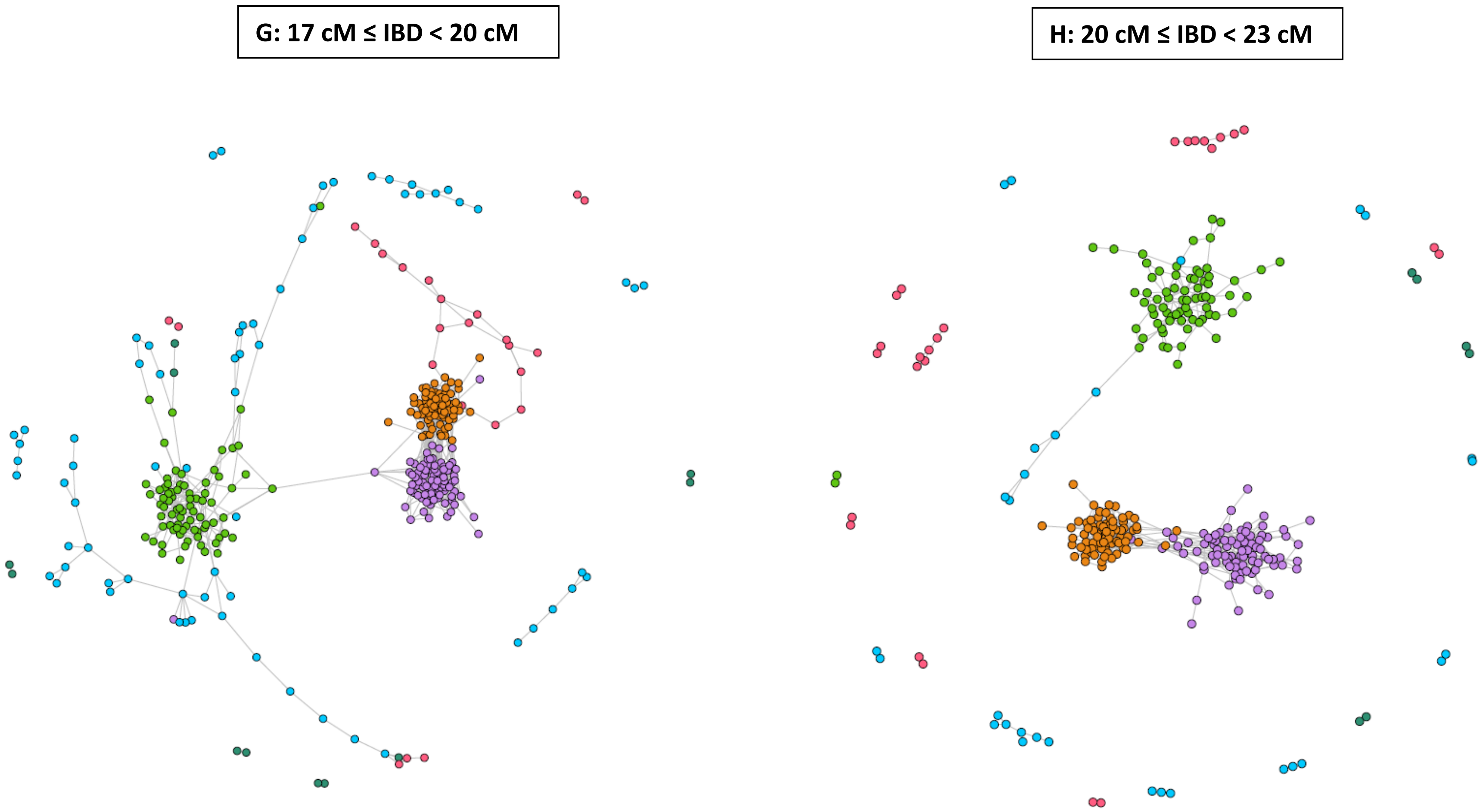

Supplementary Figure 3 (continuation)

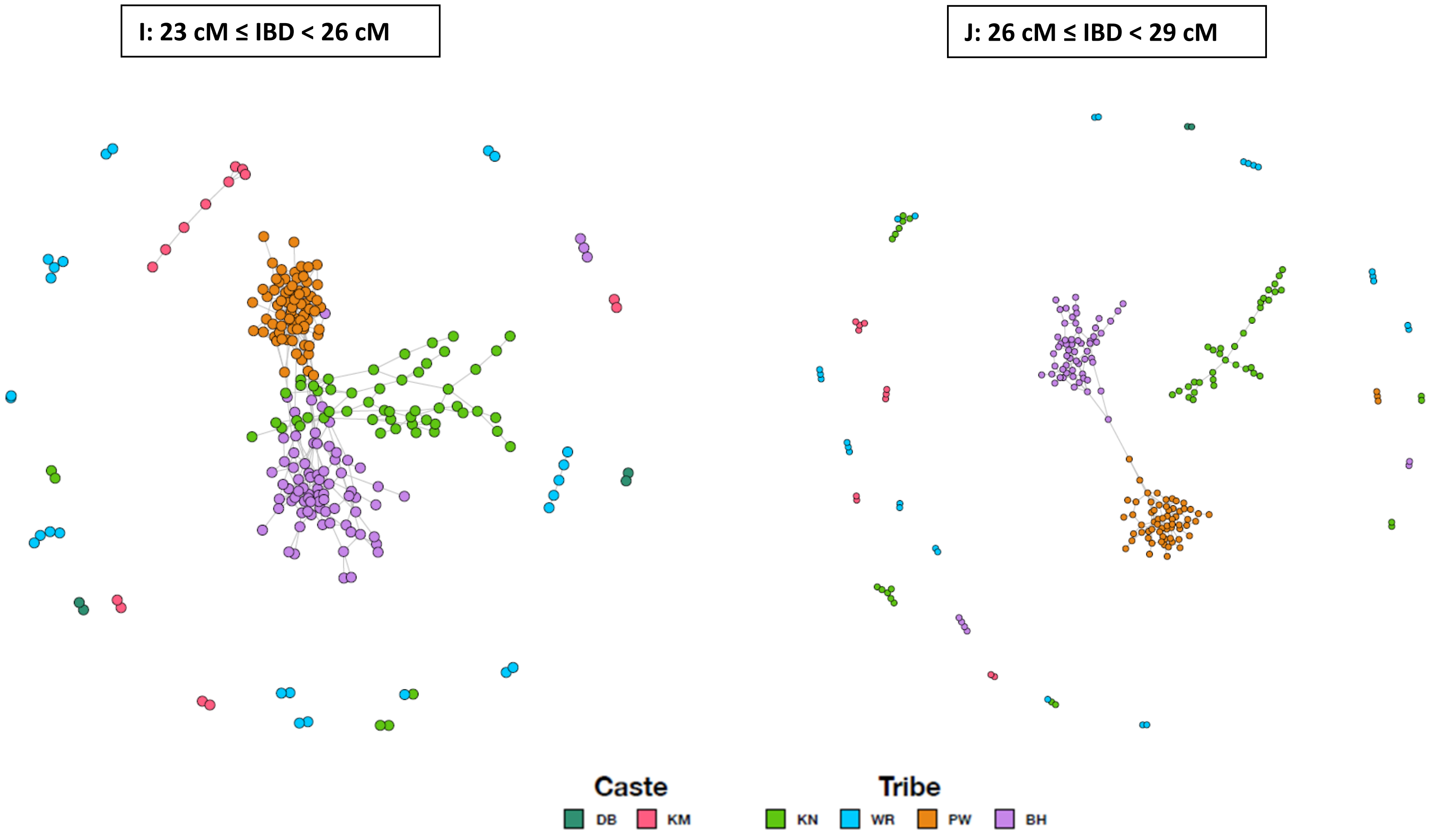

Supplementary Figure 4: Estimates of effective population sizes based on distribution of IBD segments. BH: Bhil, DB: Deshastha Bramins, KM: Kunbi Marathas, KN: Kokana, PW: Pawara, and WR: Warli.

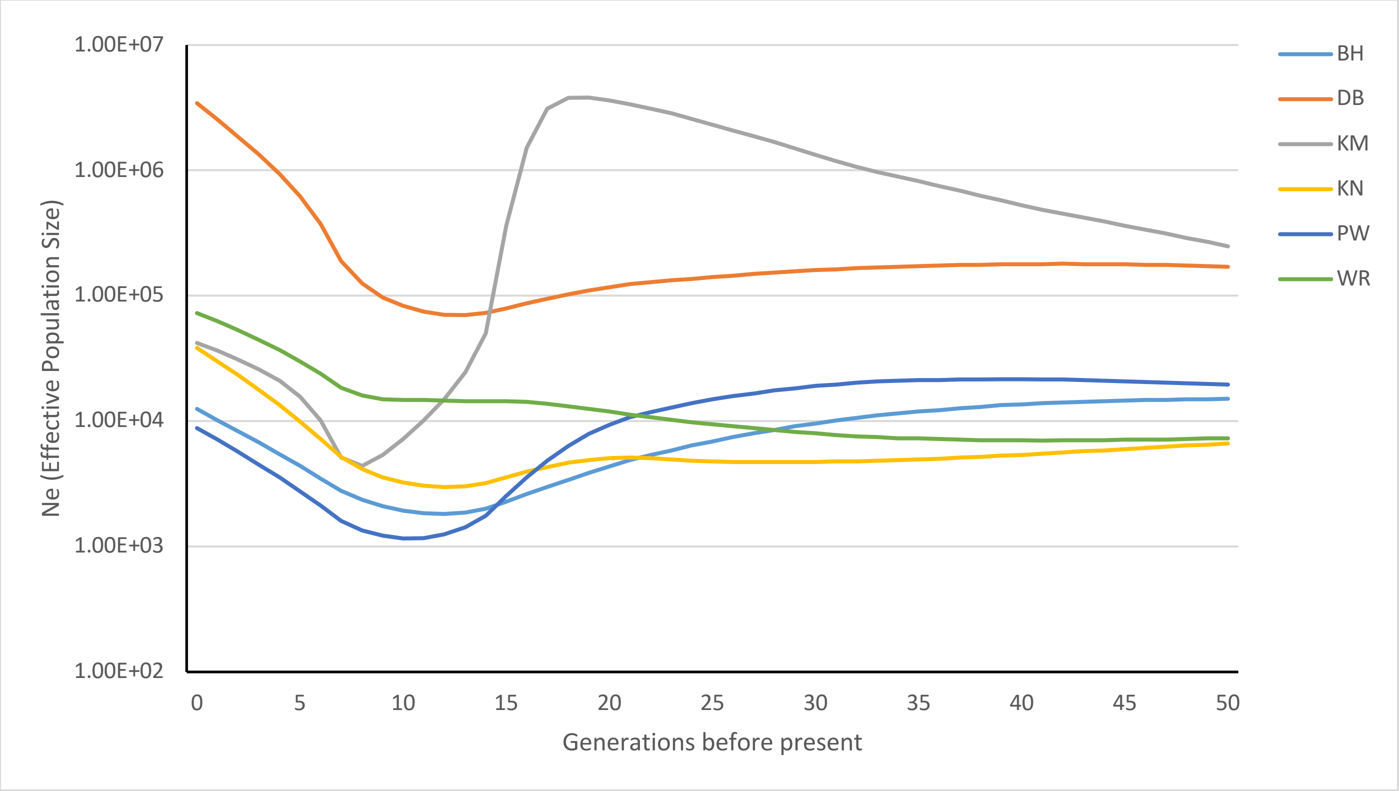

Supplementary Figure 5: PCA plot of WM tribal and caste samples, South Asian 1000 Genomes Project samples, and Indian tribal and caste groups from the SGDP.

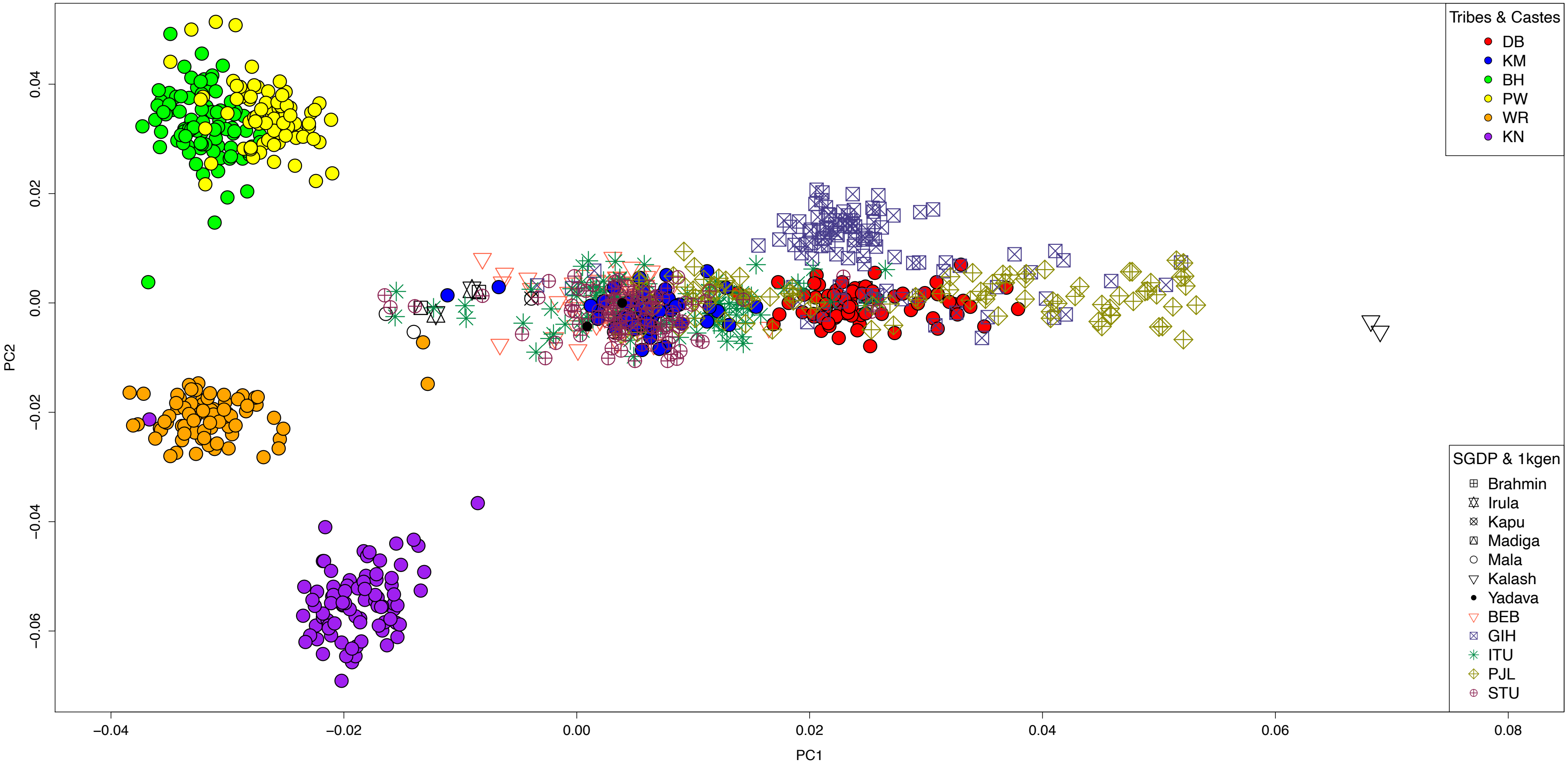

Supplementary Figure 6: Neighbor-Joining tree including the WM samples with the 1KG South Asian samples, using an African sample as an outgroup.

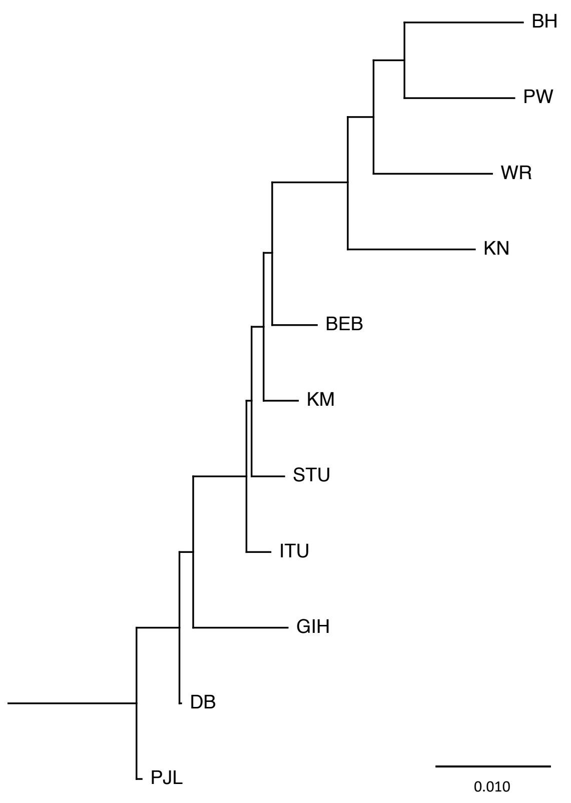

Supplementary Figure 7: PCA plot of WM tribal and caste samples, South Asian 1000 Genomes Project samples, Indian tribal and caste groups from the SGDP, and ancient DNA samples. In this plot, the ancient DNA samples were not projected onto the modern samples. For this PCA analysis, we only included markers with more than 80% genotyping rate and individuals with more than 80% genotyping rate (except one relevant sample from the Indus periphery with a slightly lower genotyping rate).

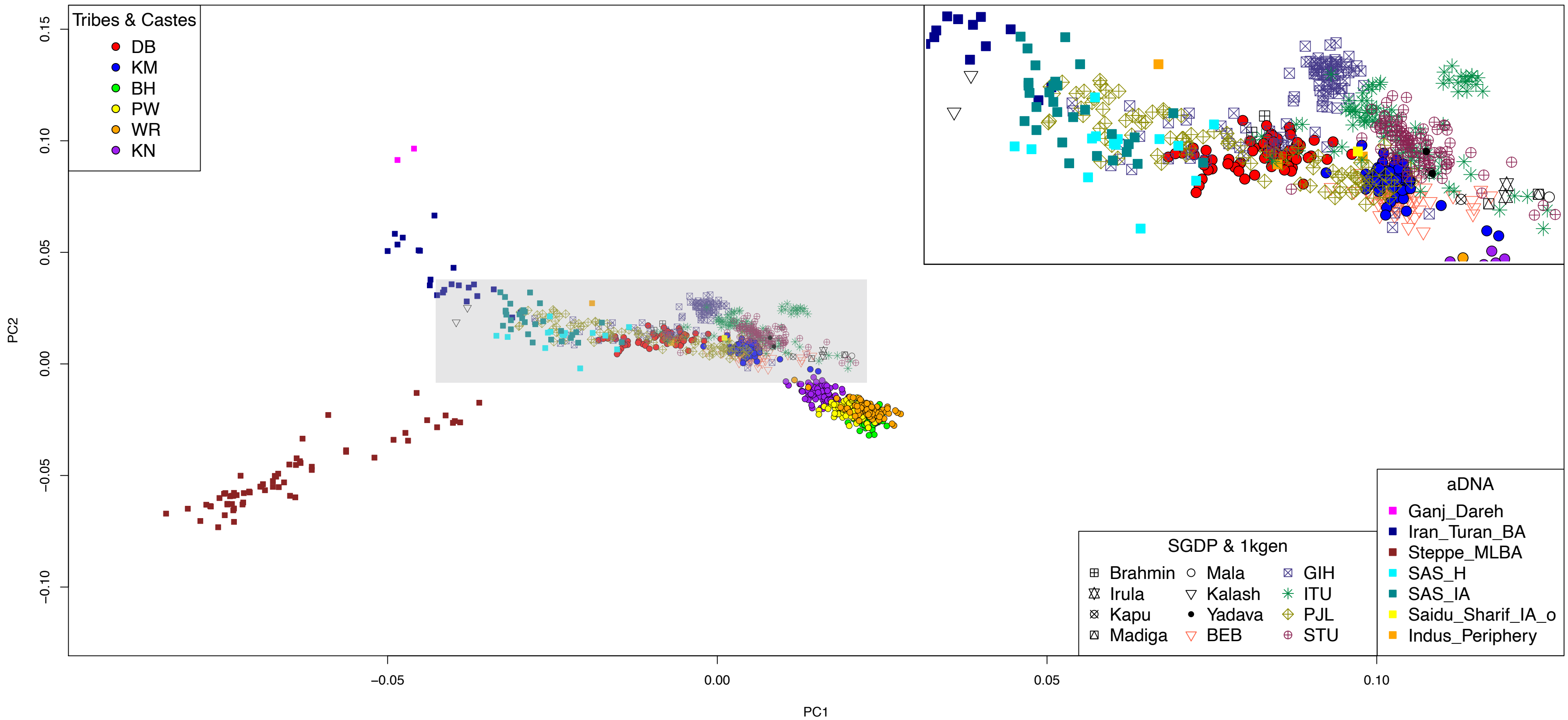

Supplementary Figure 8: Unsupervised clustering analysis using the program ADMIXTURE using K=2, 3 and 4, including tribal and caste groups from West Maharashtra, as well as relevant modern and ancient DNA samples and Andamanese samples (Onge and Jarawa). Visualization made with the R package BITE[2].

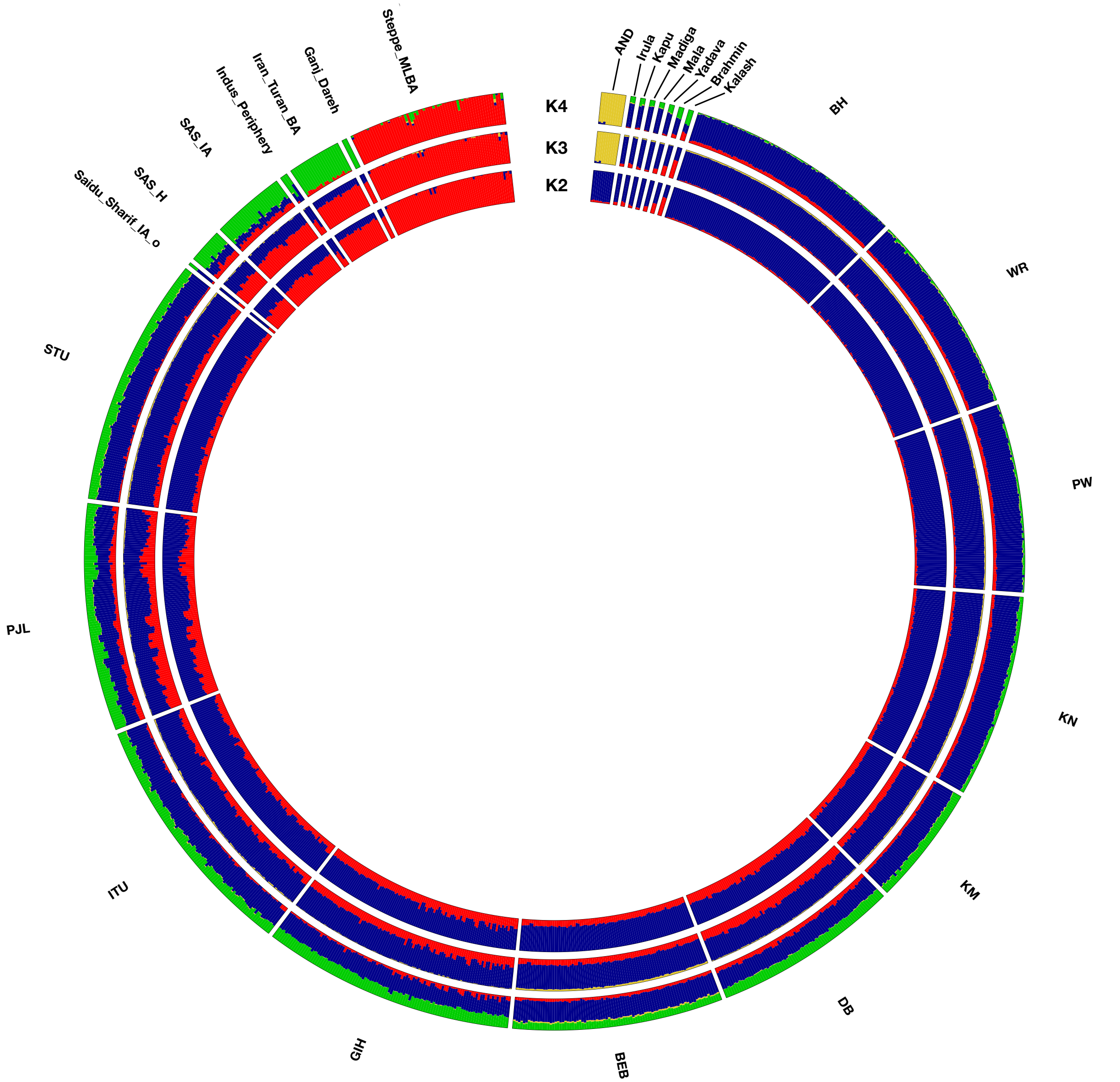

Supplementary Figure 9. f3(Test, AND; MBUTI) plot

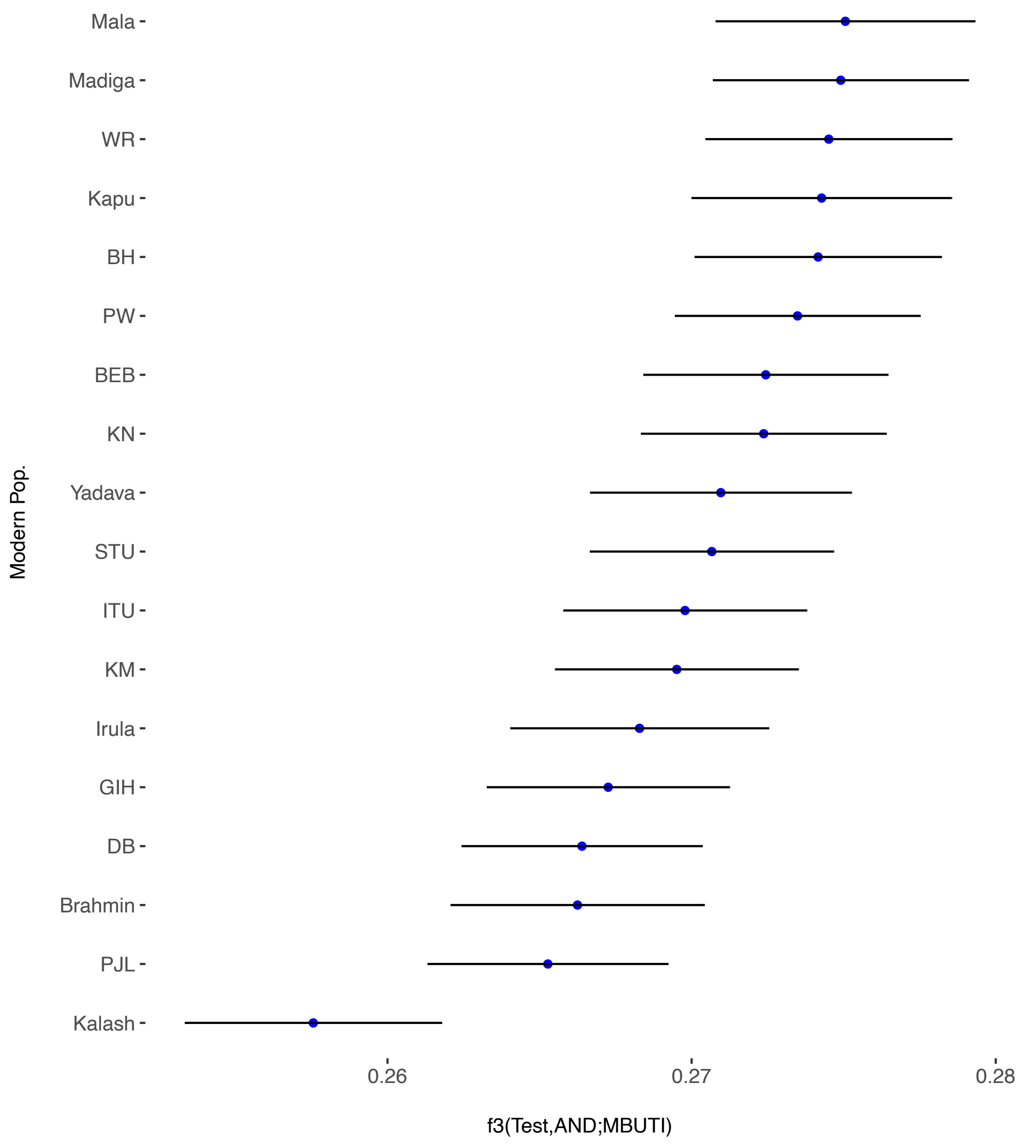

Supplementary Figure 10. f3(Test, Ganj_Dareh; MBUTI) plot

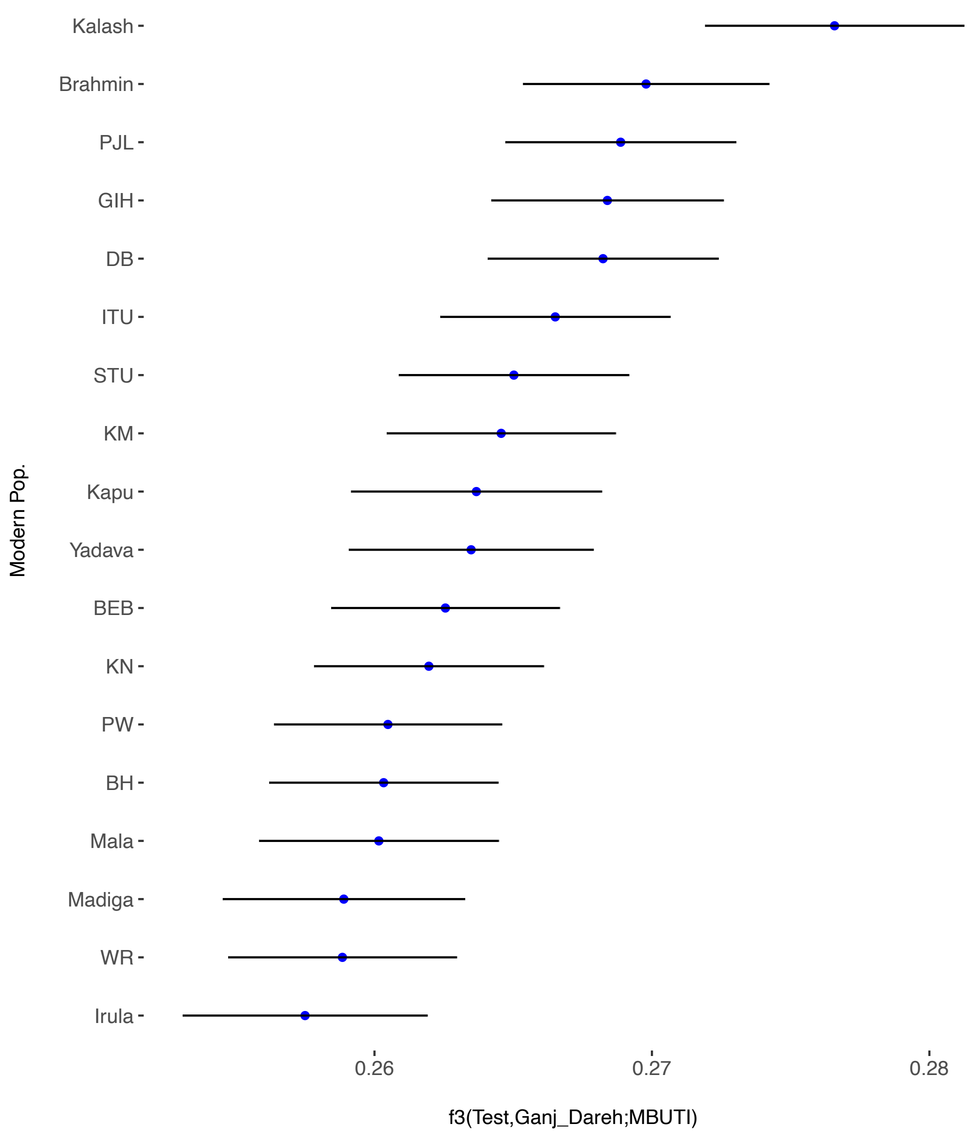

Supplementary Figure 11. f3(Test, Steppe_MLBA; MBUTI) plot

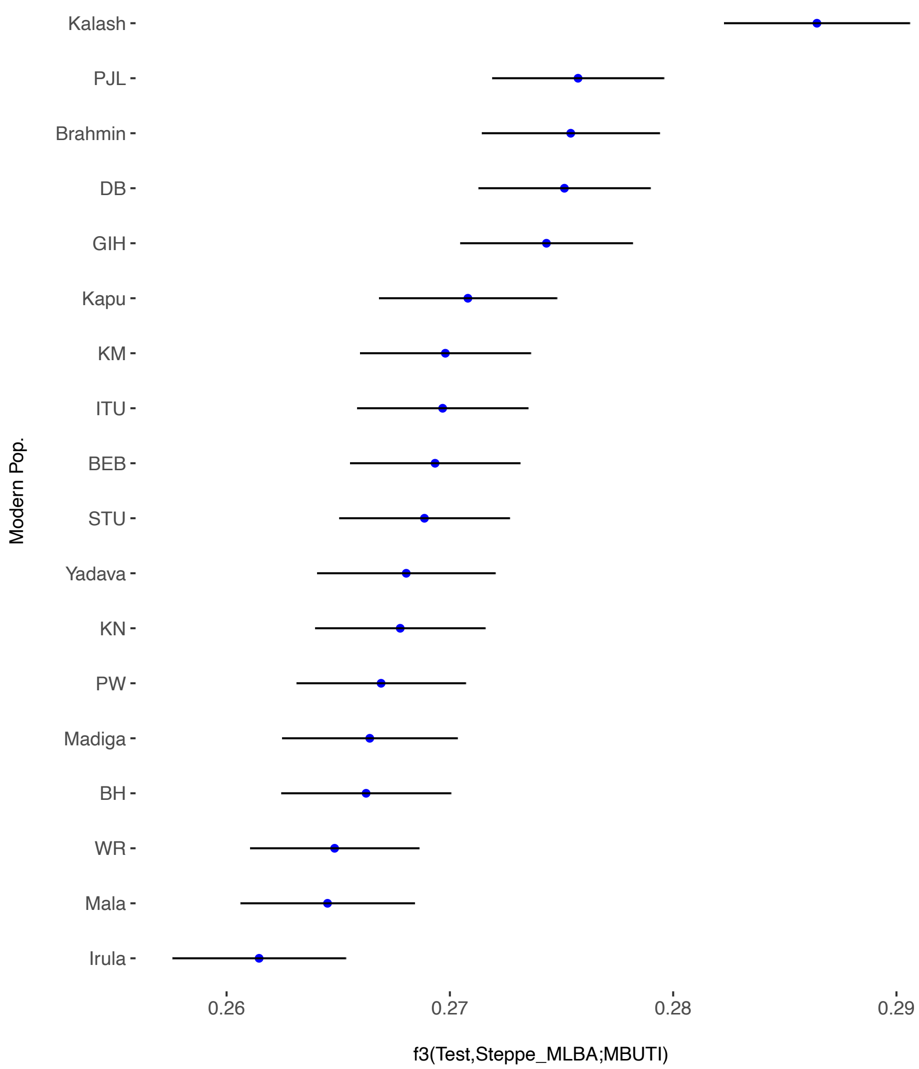

Supplementary Figure 12. f4(MBUTI, Test; AND, Ganj_Dareh) plot

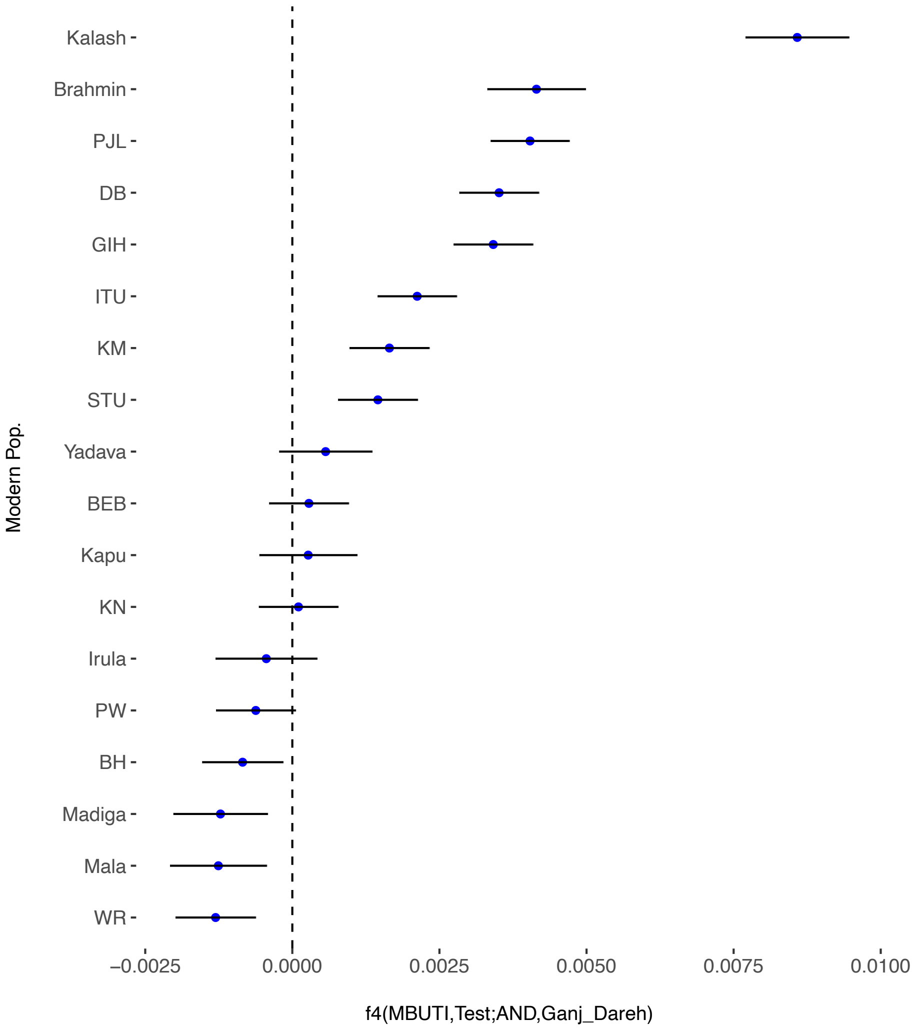

Supplementary Figure 13. f4(MBUTI, Test; AND, Steppe_MLBA) plot

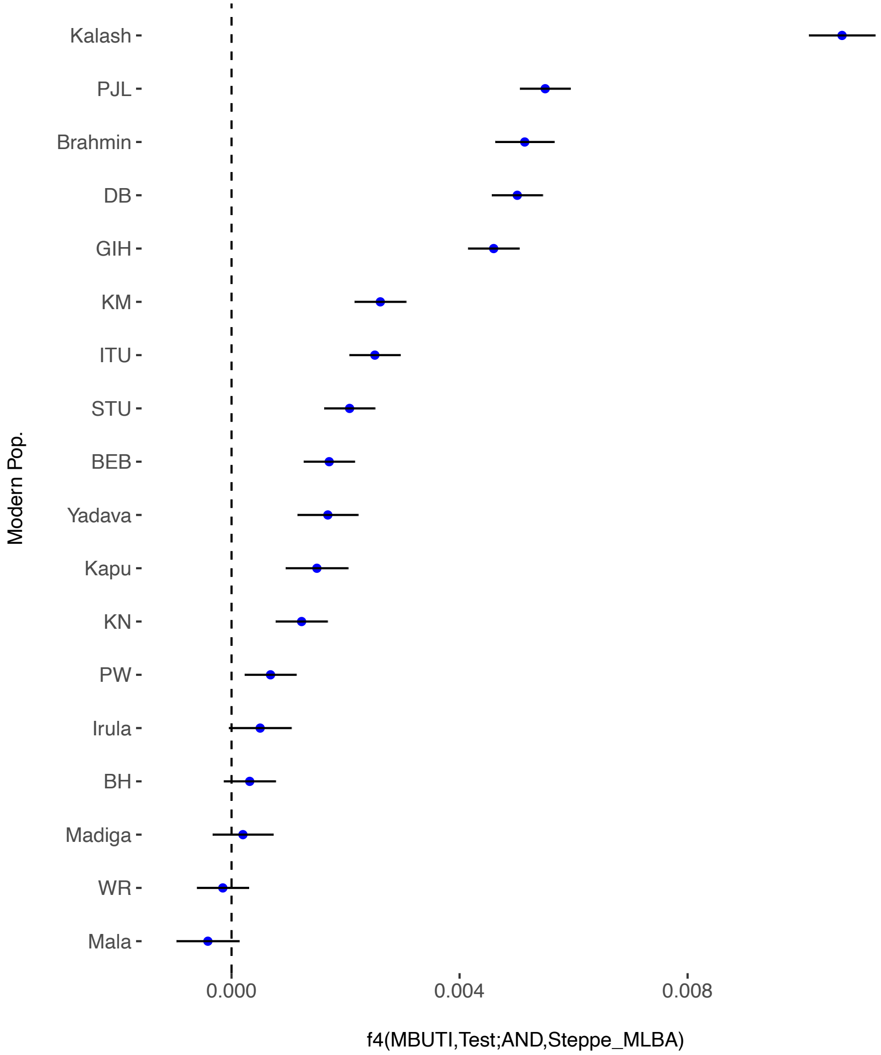

Supplementary Figure 14. f4(MBUTI, Test; Ganj_Dareh, Steppe_MLBA) plot

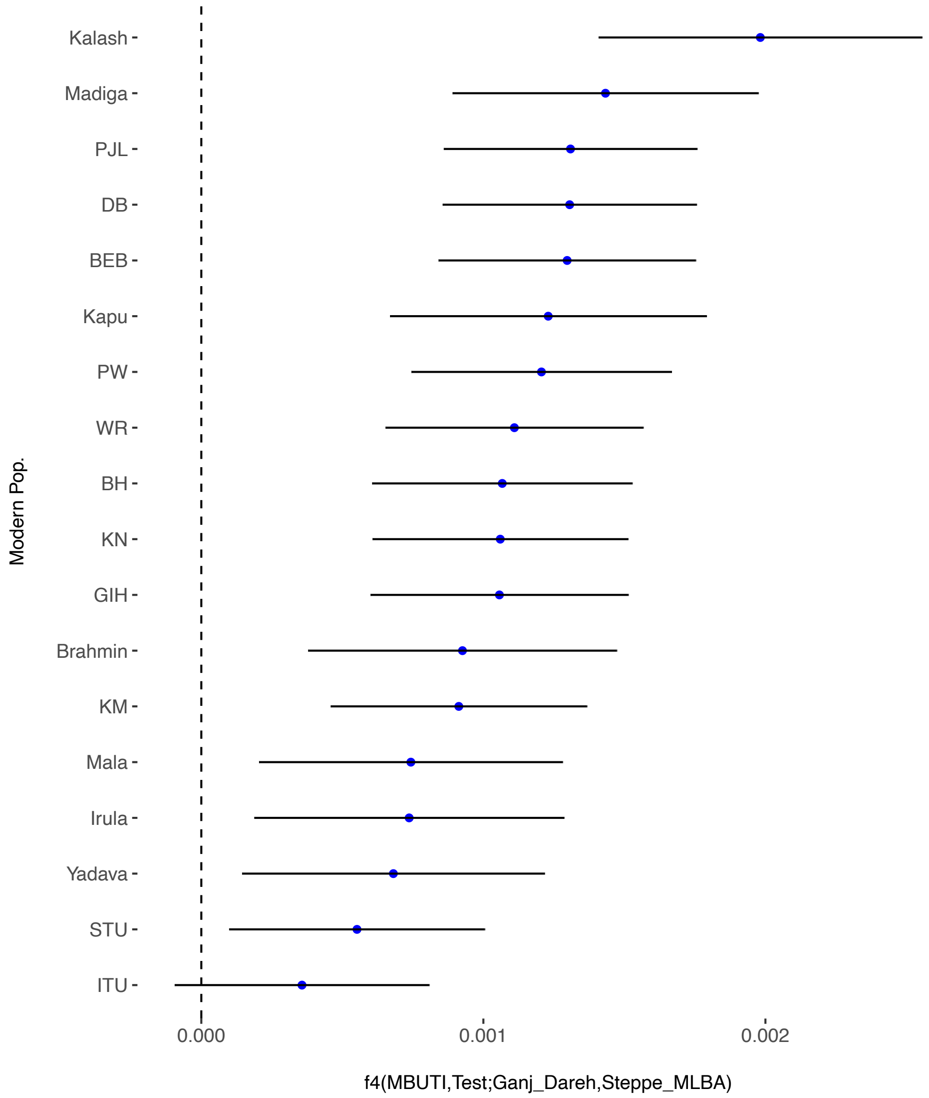

Supplementary table 1. Average and median sum of total ROH, as well as ROH above and below 1.5Mb

pop		TOT Sum ROH		SUM ROH < 1.5Mb			SUM ROH > 1.5Mb		
	N	mean	median	mean	sd	median	mean	sd	median
BH	89	352,031	344,413	294,524	13,116	296,699	57,507	38,587	47,714
KN	83	331,682	330,684	284,492	18,024	287,869	47,189	24,348	42,814
PW	77	331,030	328,700	287,975	14,032	289,647	43,055	16,047	39,054
WR	82	335,596	333,051	292,152	15,878	294,285	43,444	28,690	38,766
KM	49	331,159	309,419	279,157	14,587	282,777	52,002	72,981	26,642
DB	76	308,786	300,662	274,360	10,067	274,299	34,426	43,027	26,362
BEB	86	318,506	311,082	284,216	11,464	283,804	34,290	23,929	27,278
GIH	103	338,411	335,702	291,182	13,242	290,576	47,229	16,419	45,126
ITU	102	362,207	331,333	293,415	19,223	291,584	68,792	80,630	39,749
STU	102	370,769	344,752	284,126	14,130	285,544	86,643	78,514	59,209
PJL	93	364,595	329,844	277,840	15,863	279,488	86,755	97,228	50,356
ASW	49	84,156	81,660	78,769	8,257	77,035	5,386	3,801	4,625
ACB	72	87,543	86,979	80,997	8,508	81,246	6,546	5,871	5,734

Supplementary table 2. Summary statistics for the genomic inbreeding coefficient from ROH (FROH) across populations. FROH > 2-C: number and percentage of population with an FROH higher than a second cousin offspring. FROH > 1-C: number and percentage of population with an FROH higher than a first cousin offspring. FROH > U-N: number and percentage of population with an FROH higher than uncle - niece offspring.

pop	FROH		FROH max	FROH > 2-C		FROH >1-C		FROH >U-N	
	mean	median		N	%	N	%	N	%
BH	0.0200	0.0166	0.0958	85	95.5	5	5.6	1	1.1
KN	0.0164	0.0149	0.0598	77	92.8	3	3.6	0	0.0
PW	0.0149	0.0136	0.0325	69	89.6	0	0.0	0	0.0
WR	0.0151	0.0135	0.0832	72	87.8	2	2.4	0	0.0
KM	0.0180	0.0092	0.1287	27	55.1	5	10.2	1	2.0
DB	0.0119	0.0092	0.1137	41	53.9	2	2.6	1	1.3
BEB	0.0119	0.0095	0.0581	49	57.0	2	2.3	0	0.0
GIH	0.0164	0.0157	0.0441	99	96.1	1	1.0	0	0.0
ITU	0.0239	0.0138	0.1746	92	90.2	12	11.8	4	3.9
STU	0.0301	0.0206	0.1485	87	85.3	25	24.5	4	3.9
PJL	0.0301	0.0175	0.1497	76	81.7	21	22.6	7	7.5
ASW	0.0019	0.0016	0.0069	0	0.0	0	0.0	0	0.0
ACB	0.0023	0.0020	0.0154	1	1.4	0	0.0	0	0.0

Supplementary Table 3. Relative admixture proportions from AASI-related groups, Steppe-MLBA-related groups and ancient-Iranians (Ganj_Dareh) estimated in the WM tribal and caste groups, as well as other modern and ancient populations from South Asia, inferred with the program ADMIXTURE.

Pop	AASI	Agriculturalists	Steppe
BEB	0.7191	0.2098	0.0711
BH	0.9180	0.0132	0.0688
Brahmin	0.5576	0.3431	0.0993
DB	0.5759	0.3068	0.1173
Ganj_Dareh	0.0000	1.0000	0.0000
GIH	0.5884	0.3492	0.0624
Indus_Periphery	0.3885	0.6115	0.0000
Iran_Turan_BA	0.0000	0.9603	0.0397
Irula	0.8145	0.1809	0.0046
ITU	0.6956	0.2804	0.0240
Kalash	0.2662	0.4878	0.2460
Kapu	0.7607	0.2020	0.0372
KM	0.7074	0.2328	0.0598
KN	0.8393	0.0892	0.0715
Madiga	0.8244	0.1652	0.0104
Mala	0.8515	0.1485	0.0000
PJL	0.5434	0.3263	0.1302
PW	0.8878	0.0367	0.0755
Saidu_Sharif_IA_o	0.6429	0.3379	0.0192
SAS_H	0.3338	0.4916	0.1746
SAS_IA	0.2279	0.6275	0.1446
Steppe_MLBA	0.0108	0.0282	0.9610
STU	0.7123	0.2665	0.0212
WR	0.9156	0.0259	0.0585
Yadava	0.7322	0.2469	0.0209

Supplementary Table 4. f3 statistics, standard errors and Z-scores for f3(Test, AND; MBUTI).

	A	B	C	f3	stderr	Zscore	nsnps
1	BEB	AND	MBUTI	0.272438	0.004022	67.734	51174
2	BH	AND	MBUTI	0.274159	0.004059	67.539	51136
3	Brahmin	AND	MBUTI	0.266248	0.004173	63.803	50232
4	DB	AND	MBUTI	0.266394	0.003957	67.318	51178
5	GIH	AND	MBUTI	0.267258	0.003993	66.93	51174
6	Irula	AND	MBUTI	0.268288	0.004249	63.146	50180
7	ITU	AND	MBUTI	0.269783	0.004003	67.389	51152
8	Kalash	AND	MBUTI	0.25756	0.004222	61.008	50279
9	Kapu	AND	MBUTI	0.274274	0.004276	64.15	50201
10	KM	AND	MBUTI	0.269511	0.004001	67.361	51126
11	KN	AND	MBUTI	0.272369	0.004034	67.526	51136
12	Madiga	AND	MBUTI	0.274903	0.004206	65.358	50151
13	Mala	AND	MBUTI	0.275054	0.004263	64.514	50166
14	PJL	AND	MBUTI	0.265273	0.003955	67.073	51173
15	PW	AND	MBUTI	0.273483	0.004035	67.783	51139
16	STU	AND	MBUTI	0.270662	0.00401	67.491	51154
17	WR	AND	MBUTI	0.27451	0.004054	67.71	51130
18	Yadava	AND	MBUTI	0.27096	0.004297	63.053	50156

Supplementary Table 5. f3 statistics, standard errors and Z-scores for f3(Test, Ganj_Dareh; MBUTI).

	A	B	C	f3	stderr	Zscore	nsnps
1	BEB	Ganj_Dareh	MBUTI	0.262556	0.004117	63.766	62324
2	BH	Ganj_Dareh	MBUTI	0.260331	0.004126	63.097	61945
3	Brahmin	Ganj_Dareh	MBUTI	0.269789	0.004434	60.85	52054
4	DB	Ganj_Dareh	MBUTI	0.268237	0.004156	64.539	62372
5	GIH	Ganj_Dareh	MBUTI	0.268395	0.004184	64.146	62304
6	Irula	Ganj_Dareh	MBUTI	0.257498	0.004407	58.432	51964
7	ITU	Ganj_Dareh	MBUTI	0.266516	0.004144	64.31	62228
8	Kalash	Ganj_Dareh	MBUTI	0.276582	0.004664	59.301	51628
9	Kapu	Ganj_Dareh	MBUTI	0.263673	0.004517	58.379	52111
10	KM	Ganj_Dareh	MBUTI	0.264567	0.004124	64.16	61872
11	KN	Ganj_Dareh	MBUTI	0.26196	0.004137	63.321	61992
12	Madiga	Ganj_Dareh	MBUTI	0.258894	0.004359	59.388	52061
13	Mala	Ganj_Dareh	MBUTI	0.260157	0.004316	60.276	52092
14	PJL	Ganj_Dareh	MBUTI	0.268873	0.004153	64.748	62397
15	PW	Ganj_Dareh	MBUTI	0.260485	0.004105	63.462	61923
16	STU	Ganj_Dareh	MBUTI	0.265024	0.004147	63.913	62203
17	WR	Ganj_Dareh	MBUTI	0.258845	0.004116	62.891	61990
18	Yadava	Ganj_Dareh	MBUTI	0.263484	0.004406	59.806	51922

Supplementary Table 6. f3 statistics, standard errors and Z-scores for f3(Test, Steppe_MLBA; MBUTI).

	A	B	C	f3	stderr	Zscore	nsnps
1	BEB	Steppe_MLBA	MBUTI	0.269338	0.003807	70.748	68924
2	BH	Steppe_MLBA	MBUTI	0.266248	0.003797	70.121	68820
3	Brahmin	Steppe_MLBA	MBUTI	0.275413	0.003979	69.223	66467
4	DB	Steppe_MLBA	MBUTI	0.27513	0.003849	71.478	68914
5	GIH	Steppe_MLBA	MBUTI	0.274325	0.003858	71.114	68858
6	Irula	Steppe_MLBA	MBUTI	0.261457	0.003881	67.37	66515
7	ITU	Steppe_MLBA	MBUTI	0.269676	0.003826	70.489	68874
8	Kalash	Steppe_MLBA	MBUTI	0.286438	0.004156	68.922	66317
9	Kapu	Steppe_MLBA	MBUTI	0.270808	0.003983	67.992	66550
10	KM	Steppe_MLBA	MBUTI	0.269798	0.003822	70.582	68730
11	KN	Steppe_MLBA	MBUTI	0.267776	0.003808	70.318	68794
12	Madiga	Steppe_MLBA	MBUTI	0.266413	0.003924	67.885	66526
13	Mala	Steppe_MLBA	MBUTI	0.26452	0.003898	67.866	66567
14	PJL	Steppe_MLBA	MBUTI	0.275741	0.003842	71.77	68912
15	PW	Steppe_MLBA	MBUTI	0.26692	0.003787	70.488	68813
16	STU	Steppe_MLBA	MBUTI	0.268861	0.003814	70.498	68874
17	WR	Steppe_MLBA	MBUTI	0.264839	0.003782	70.025	68843
18	Yadava	Steppe_MLBA	MBUTI	0.268046	0.003988	67.208	66567

Supplementary Table 7. f4 statistics, standard errors and Z-scores for f4(MBUTI, Test; AND,Ganj_Dareh).

	W	X	Y	Z	f4	stderr	Zscore	BABA	ABBA	nsnps
1	MBUTI	BEB	AND	Ganj_Dareh	0.000282	0.000675	0.418	3902	3889	46311
2	MBUTI	BH	AND	Ganj_Dareh	-0.00085	0.000686	-1.231	3875	3914	46311
3	MBUTI	Brahmin	AND	Ganj_Dareh	0.004149	0.000834	4.977	3983	3791	46311
4	MBUTI	DB	AND	Ganj_Dareh	0.003514	0.000675	5.205	3970	3807	46311
5	MBUTI	GIH	AND	Ganj_Dareh	0.003415	0.000673	5.077	3969	3811	46311
6	MBUTI	Irula	AND	Ganj_Dareh	-0.00044	0.000861	-0.514	3872	3893	46311
7	MBUTI	ITU	AND	Ganj_Dareh	0.002122	0.000671	3.162	3941	3843	46311
8	MBUTI	Kalash	AND	Ganj_Dareh	0.008581	0.000878	9.771	4089	3692	46311
9	MBUTI	Kapu	AND	Ganj_Dareh	0.00027	0.000829	0.325	3912	3899	46311
10	MBUTI	KM	AND	Ganj_Dareh	0.00165	0.000677	2.438	3927	3850	46311
11	MBUTI	KN	AND	Ganj_Dareh	0.000105	0.000673	0.156	3893	3888	46311
12	MBUTI	Madiga	AND	Ganj_Dareh	-0.00122	8.00E-04	-1.526	3860	3916	46311
13	MBUTI	Mala	AND	Ganj_Dareh	-0.00126	0.00082	-1.534	3871	3929	46311
14	MBUTI	PJL	AND	Ganj_Dareh	0.00404	0.000668	6.049	3980	3793	46311
15	MBUTI	PW	AND	Ganj_Dareh	-0.00062	0.000676	-0.919	3879	3908	46311
16	MBUTI	STU	AND	Ganj_Dareh	0.001454	0.000675	2.153	3925	3858	46311
17	MBUTI	WR	AND	Ganj_Dareh	-0.0013	0.00068	-1.919	3863	3923	46311
18	MBUTI	Yadava	AND	Ganj_Dareh	0.000566	0.000788	0.717	3895	3869	46311

Supplementary Table 8. f4 statistics, standard errors and Z-scores for f4(MBUTI, Test; AND,Steppe_MLBA).

	W	X	Y	Z	f4	stderr	Zscore	BABA	ABBA	nsnps
1	MBUTI	BEB	AND	Steppe_MLBA	0.001713	0.000446	3.84	4338	4250	51061
2	MBUTI	BH	AND	Steppe_MLBA	0.000319	0.000453	0.703	4300	4284	51061
3	MBUTI	Brahmin	AND	Steppe_MLBA	0.005143	0.000517	9.95	4415	4152	51061
4	MBUTI	DB	AND	Steppe_MLBA	0.005012	0.000445	11.271	4415	4159	51061
5	MBUTI	GIH	AND	Steppe_MLBA	0.004598	0.000448	10.265	4406	4171	51061
6	MBUTI	Irula	AND	Steppe_MLBA	0.000502	0.000547	0.918	4275	4249	51061
7	MBUTI	ITU	AND	Steppe_MLBA	0.002514	0.000445	5.644	4352	4223	51061
8	MBUTI	Kalash	AND	Steppe_MLBA	0.010709	0.000581	18.421	4559	4012	51061
9	MBUTI	Kapu	AND	Steppe_MLBA	0.001497	0.000547	2.735	4346	4270	51061
10	MBUTI	KM	AND	Steppe_MLBA	0.00261	0.000451	5.789	4352	4218	51061
11	MBUTI	KN	AND	Steppe_MLBA	0.001229	0.000454	2.709	4322	4259	51061
12	MBUTI	Madiga	AND	Steppe_MLBA	2.00E-04	0.00053	0.378	4301	4291	51061
13	MBUTI	Mala	AND	Steppe_MLBA	-0.00042	0.00055	-0.754	4277	4298	51061
14	MBUTI	PJL	AND	Steppe_MLBA	0.005502	0.000441	12.472	4426	4145	51061
15	MBUTI	PW	AND	Steppe_MLBA	0.000684	0.000452	1.513	4309	4274	51061
16	MBUTI	STU	AND	Steppe_MLBA	0.002071	0.000445	4.654	4340	4235	51061
17	MBUTI	WR	AND	Steppe_MLBA	-0.00015	0.000453	-0.337	4287	4295	51061
18	MBUTI	Yadava	AND	Steppe_MLBA	0.001689	0.000531	3.183	4320	4234	51061

Supplementary Table 9. f4 statistics, standard errors and Z-scores for f4(MBUTI, Test; Ganj_Dareh,Steppe_MLBA).

	W	X	Y	Z	f4	stderr	Zscore	BABA	ABBA	nsnps
1	MBUTI	BEB	Ganj_Dareh	Steppe_MLBA	0.001297	0.000456	2.847	4123	4041	62732
2	MBUTI	BH	Ganj_Dareh	Steppe_MLBA	0.001067	0.000461	2.315	4107	4040	62732
3	MBUTI	Brahmin	Ganj_Dareh	Steppe_MLBA	0.000926	0.000547	1.692	4129	4071	62732
4	MBUTI	DB	Ganj_Dareh	Steppe_MLBA	0.001306	0.00045	2.902	4141	4059	62732
5	MBUTI	GIH	Ganj_Dareh	Steppe_MLBA	0.001057	0.000457	2.314	4132	4066	62732
6	MBUTI	Irula	Ganj_Dareh	Steppe_MLBA	0.000737	0.000549	1.342	4082	4036	62732
7	MBUTI	ITU	Ganj_Dareh	Steppe_MLBA	0.000357	0.000451	0.791	4098	4076	62732
8	MBUTI	Kalash	Ganj_Dareh	Steppe_MLBA	0.001982	0.000573	3.457	4197	4073	62732
9	MBUTI	Kapu	Ganj_Dareh	Steppe_MLBA	0.00123	0.000561	2.193	4125	4048	62732
10	MBUTI	KM	Ganj_Dareh	Steppe_MLBA	0.000913	0.000454	2.011	4111	4054	62732
11	MBUTI	KN	Ganj_Dareh	Steppe_MLBA	0.00106	0.000453	2.341	4110	4043	62732
12	MBUTI	Madiga	Ganj_Dareh	Steppe_MLBA	0.001433	0.000542	2.643	4116	4026	62732
13	MBUTI	Mala	Ganj_Dareh	Steppe_MLBA	0.000743	0.000538	1.382	4088	4041	62732
14	MBUTI	PJL	Ganj_Dareh	Steppe_MLBA	0.001309	0.000449	2.912	4143	4061	62732
15	MBUTI	PW	Ganj_Dareh	Steppe_MLBA	0.001206	0.000461	2.618	4113	4038	62732
16	MBUTI	STU	Ganj_Dareh	Steppe_MLBA	0.000552	0.000453	1.218	4101	4066	62732
17	MBUTI	WR	Ganj_Dareh	Steppe_MLBA	0.00111	0.000457	2.432	4102	4032	62732
18	MBUTI	Yadava	Ganj_Dareh	Steppe_MLBA	0.000681	0.000536	1.271	4095	4053	62732

Supplementary Table 10. Relative admixture proportions from AASI-related groups (Onge: ONG), Steppe-MLBA-related groups (STP_MLBA) and Indus-Periphery related groups (IND-P) estimated in the WM tribal and caste groups, as well as other populations from South Asia. Admixture proportions were estimated with the program qpAdm, using Onge, Steppe-MLBA and Indus Periphery as source groups, and Mbuti, Iron_Gates_HG, Karelia_HG, Ganj_Dareh_N, Anatolia_N, West_Siberia_N, Han, Karitiana as outgroups. The table also reports standard error of admixture estimates and the P-value of the model.

target	IND-P	ONG	STP_MLBA	se-IND-P	se-ONG	se-STP_MLBA	p-value
Mala	0.506	0.472	0.022	0.064	0.038	0.039	0.577
Irula	0.459	0.497	0.044	0.064	0.037	0.039	0.093
ITU	0.604	0.335	0.061	0.047	0.027	0.028	0.019
Madiga	0.426	0.508	0.066	0.057	0.034	0.036	0.775
Kapu	0.501	0.433	0.066	0.061	0.036	0.038	0.043
STU	0.555	0.376	0.069	0.045	0.026	0.027	0.017
BH	0.43	0.498	0.072	0.041	0.024	0.025	0.062
BEB	0.412	0.515	0.073	0.038	0.023	0.023	0.028
WR	0.383	0.54	0.077	0.04	0.024	0.024	0.095
PW	0.431	0.48	0.088	0.039	0.024	0.023	0.093
KN	0.459	0.449	0.091	0.041	0.024	0.024	0.076
Yadava	0.478	0.421	0.102	0.062	0.038	0.036	0.067
KM	0.527	0.37	0.103	0.042	0.025	0.026	0.035
GIH	0.591	0.266	0.143	0.045	0.026	0.027	0.02
Brahmin	0.601	0.251	0.148	0.062	0.038	0.037	0.113
DB	0.558	0.264	0.178	0.044	0.025	0.027	0.008
PJL	0.567	0.242	0.191	0.044	0.025	0.026	0.01

References:

1. Bastian, M., Heymann, S. & Jacomy, M. Gephi: an open source software for exploring and manipulating networks. in International AAAI Conference on Weblogs and Social Media, (2009).
2. Milanesi, M. *et al.* BITE: an R package for biodiversity analyses. *bioRxiv* (2017).
